# Synthetic Pathways to Non-Psychotropic Phytocannabinoids as Promising Molecules to Develop Novel Antibiotics: A Review

**DOI:** 10.3390/pharmaceutics15071889

**Published:** 2023-07-05

**Authors:** Silvana Alfei, Gian Carlo Schito, Anna Maria Schito

**Affiliations:** 1 Department of Pharmacy (DIFAR), University of Genoa, Viale Cembrano, 4, 16148 Genoa, Italy; 2 Department of Surgical Sciences and Integrated Diagnostics (DISC), University of Genoa, Viale Benedetto XV, 6, 16132 Genova, Italy; giancarlo.schito@unige.it (G.C.S.); amschito@unige.it (A.M.S.)

**Keywords:** bacterial resistance, methicillin-resistant *S. aureus* (MRSA), multi drug resistant (MDR) bacteria, *Cannabis sativa*, phytocannabinoids (PCs), endocannabinois (ECs), synthetic cannabinoids (SCs), synthetic procedures, cannabichromene (CBC), cannabigerol (CBG), cannabidiol (CBC)

## Abstract

Due to the rapid emergence of multi drug resistant (MDR) pathogens against which current antibiotics are no longer functioning, severe infections are becoming practically untreatable. Consequently, the discovery of new classes of effective antimicrobial agents with novel mechanism of action is becoming increasingly urgent. The bioactivity of *Cannabis sativa*, an herbaceous plant used for millennia for medicinal and recreational purposes, is mainly due to its content in phytocannabinoids (PCs). Among the 180 PCs detected, cannabidiol (CBD), Δ^8^ and Δ^9^-tetrahydrocannabinols (Δ^8^-THC and Δ^9^-THC), cannabichromene (CBC), cannabigerol (CBG), cannabinol (CBN) and some of their acidic precursors have demonstrated from moderate to potent antibacterial effects against Gram-positive bacteria (MICs 0.5–8 µg/mL), including methicillin-resistant *Staphylococcus aureus* (MRSA), epidemic MRSA (EMRSA), as well as fluoroquinolone and tetracycline-resistant strains. Particularly, the non-psychotropic CBG was also capable to inhibit MRSA biofilm formation, to eradicate even mature biofilms, and to rapidly eliminate MRSA persiter cells. In this scenario, CBG, as well as other minor non-psychotropic PCs, such as CBD, and CBC could represent promising compounds for developing novel antibiotics with high therapeutic potential. Anyway, further studies are necessary, needing abundant quantities of such PCs, scarcely provided naturally by *Cannabis* plants. Here, after an extensive overture on cannabinoids including their reported antimicrobial effects, aiming at easing the synthetic production of the necessary amounts of CBG, CBC and CBD for further studies, we have, for the first time, systematically reviewed the synthetic pathways utilized for their synthesis, reporting both reaction schemes and experimental details.

## 1. Introduction

**Figure 1:**
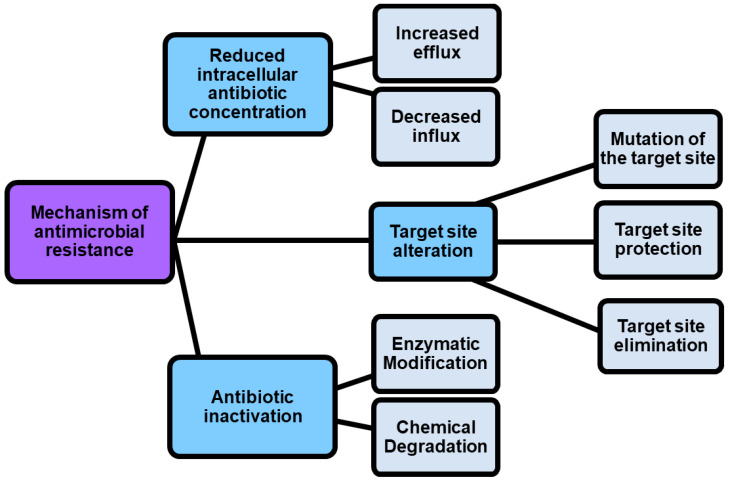
Mechanisms by which pathogens can become resistant.

Given the rapid emergence of multi drug resistant (MDR), extensively drug-resistant (XDR) and pandrug-resistant (PDR) pathogens, against which current antibiotics are no longer functioning, we are rapidly moving into a post-antibiotic era where infections will be practically untreatable [1]. According to the definition of the World Health Organization (WHO), antimicrobial resistance is a natural event that occurs when microbes become tolerant to drugs originally active, thus rendering several infections more difficult or impossible to treat [2,3]. Particularly, WHO has identified twelve families of bacteria to be considered as the most dangerous to human health. These families have been assigned to three priority groups, comprising critical pathogens (*Acinetobacter*, *Pseudomonas*, and *Enterobacteriaceae*), high priority pathogens (*Enterococcus faecium*, *Staphylococcus aureus*, *Helicobacter pylori*, *Campylobacter*, *Salmonella* spp., and *Neisseria gonorrhoeae*), and medium priority pathogens (*Streptococcus pneumoniae*, and *Shigella* spp.) [3,4]. Resistance in bacteria can be acquired or natural, but several mechanisms exist by which pathogens can become resistant to antibiotics (Figure 1).

As shown in Figure 1, antimicrobial resistance mechanisms include drug inactivation, decreased intracellular drug concentration, and altered drug targets [5]. 

### 1.1. Mechanism of Antimicrobial Resistance vs. Strategies to Develop Novel Antibiotics

Drug inactivation can occur either by enzymatic or chemical degradation, while decreased intracellular drug concentration can occur because of increasing drugs efflux and decreasing drugs influx [5]. In this regard, porin mutations in resistant strains alter the permeability of bacterial membranes, thus reducing the uptake of antibiotics into the bacterial cell. On the contrary, the hyperexpression of efflux pumps, which pump antibiotics out of the cell, dramatically reduces their concentration inside the cell [6]. Also, by the action of enzymes that chemically modify components of the bacterial outer membrane essential for antibiotic binding, some Gram-negative bacteria such as *P. aeruginosa*, *Acinetobacter baumannii* and others develop resistance to glycopeptide and polymyxin antibiotics. Furthermore, methyltransferases are a class of enzymes capable to modify the target thus promoting the resistance to antibiotics including aminoglycoside, lincosamide, macrolide, streptogramin, and oxazolidinone [7]. Another phenomenon known as “target protection” occurs when antibiotic target’s resistance proteins, such as the tetracycline ribosomal protection proteins (TRPPs), protect bacteria from the antibiotic-induced inhibition [8]. Additionally, the antibiotic resistance could be caused by the use of antibiotics in feed diet for animal production. The overuse, abuse, and misuse of β-lactams, aminoglycosides, tetracyclines, macrolides, and other antibiotics, with the purpose of promoting the development of animals, can cause the presence of residual antibiotics in the products intended for human consumption obtained from those animals, and can determine antibiotics pollution into the environment [9,10,11]. It was reported that some bacterial infections in humans are sustained by animal pathogens, namely zoonotic pathogens, thus proving that antibiotic resistance can be directly or indirectly transmitted from animal to humans [9]. A few practices, including the improvement of animal feed, waste management, and animal natural immunity, as well as the use of antibiotic alternatives such as prebiotics, probiotic vaccines, and bacteriophages can regulate and limit the antibiotic resistance, thus maintaining the potency of the available drugs [12]. However, more strategies to counteract antibiotic resistance are necessary, and currently they include the use of nanotechnology, computational methods, the use of antibiotic alternatives, drug repurposing, the synthesis of novel antibacterial agents, prodrugs, the development of efficient diagnostic agents also named rapid diagnostic tests (RDTs), the use of combination therapy, as well as the awareness, and knowledge of antibiotic prescribing (Table 1). 

### 1.2. Cannabinoids as Strategic Compounds to Develop New Antibiotics 

Omitting to comment on each strategy reported in Table 1, because it is out ofthe scope of this study, and instead focusing on the development of alternative antibiotics, we can observe that cannabinoids, better known for many other pharmacological and psychotropic effects are included in this category. Particularly, cannabinoids are prenylated polyketides produced in *Cannabis* plants and particularly in *Cannabis sativa*, which is an herbaceous plant that has been used for millennia for both medicinal and recreational purposes. *C. sativa* possesses a plethora of pharmacological properties and mind-altering effects, largely due to its content in cannabinoids, more precisely phytocannabinois (PCs), given their vegetable origin [28]. Collectively, more than 1600 chemical compounds have been isolated from *C. sativa*, of which over 500 are phytochemicals including cannabinoids, flavonoids terpenoids and sterols [28]. Among phytochemicals, more than 180 are cannabinoids, about 125 have been isolated, that can be classified into 11 structural families [28,29]. The most abundant representatives of these families are Δ^9^-tetrahydrocannabinol (Δ^9^-THC, also the main psychoactive cannabinoid), cannabidiol (CBD), and cannabichromene (CBC). Additionally, other classes whose prototypes are Δ^8^-*E*-tetrahydrocannabinol (Δ^8^-THC), cannabigerol (CBG), cannabinodiol (CBND), cannabielsoin (CBE), cannabicyclol (CBL), cannabinol (CBN), cannabitriol (CBT), and a miscellaneous group have been identified [28,29]. Currently, despite its psychotropic effects, Δ^9^- THC is used as therapeutic agent in the treatment of chemotherapy-associated nausea and vomiting, AIDS related loss of appetite, as well as pain and muscle spasms in multiple sclerosis [30]. Also, its carboxylic acid precursor, THCA, not exerting psycho-active effects in humans, is currently examined for its immunomodulatory, anti-inflammatory, neuroprotective and anti-neoplastic effects as well for its effectiveness in reducing adiposity and preventing metabolic disease caused by diet-induced obesity [31]. CBD, non-psychotropic as well, is currently investigated for application in the treatment of Alzheimer’s disease, Parkinson’s disease, epilepsy, cancer and for its neuroprotective efficacy [32]. Although the most studied cannabinoids for medicinal purposes are CBD and Δ^9^-THC, nowadays the research focus moves increasingly towards other PCs, such as the not psychoactive CBC, currently investigated for its anti-inflammatory, anti-fungal, antibiotic and analgesic effects [30], CBG and cannabigerolic acid (CBGA), which is the precursor of the decarboxylated CBG and could be considered as the “mother of all cannabinoids” (see later). Particularly, CBG has many putative benefits ranging from anti-inflammatory action to pain reliever [33]. Among other more investigated therapeutic properties, PCs including Δ^9^-THC, Δ^8^-THC, CBD, CBN, CBG, and CBC and some their correspondent carboxylic acids have shown from moderate to potent antimicrobial properties mainly against Gram-positive bacteria (MICs 0.5–8 µg/mL), and especially against strains of *S. aureus*, including MRSA, EMRSA, as well as fluoroquinolone and tetracycline-resistant strains, [34]. Particularly, even if the precise mechanisms used by PCs remains unknown so far, recent investigations have revealed that PCs inhibits bacteria by injuring their cytoplasmic membrane [35,36]. Recently, Luz-Veiga et al. have reported the antibacterial activity of both CBD and CBG, being CBG the most potent compound, and their capability to inhibit *Staphylococci* adherence to keratinocytes without compromising skin microbiota, thus being very promising as antibacterial agents to treat skin infection by topical administration [37]. Blaskovich et al., in addition to confirm the antibacterial activity of CBD on Gram-positive pathogens, including highly resistant *S. aureus*, *S. pneumoniae*, and *Clostridioides difficile*, demonstrated that CBD has excellent activity against biofilms, little propensity to induce resistance, and topical in vivo efficacy [38]. Moreover, the authors reported that CBD can selectively kill a subset of Gram-negative bacteria that includes the ‘urgent threat’ pathogen *Neisseria gonorrhoeae* [38]. Additionally, the interaction of CBD with broad-spectrum antibiotics such as ampicillin, kanamycin, and polymyxin B was studied by Gildea et al. [39]. By disrupting membrane integrity at extremely low dosages, CBD-antibiotic co-therapy showed synergistic activity against *Salmonella typhimurium*, offering an intriguing alternative in the treatment of this clinically relevant bacterium. The impressively strong antibacterial activity against MRSA of CBG has been reported by Farha et al. in the year 2020 [33]. Even in comparison with standard therapy with vancomycin, CBG outcompetes classical approaches against MRSA. Additionally, CBG demonstrated to inhibit the capability of MRSA to generate de novo biofilm, showed to succeed in disaggregating the pre-formed biofilm, to kill rapidly stationary phase cells (persisters), and to effectively inhibit MRSA also in vivo, in a murine model. The authors speculated that *C. sativa* may produce PCs as a natural defense mechanism against pathogens and suggested PCs as a new compound class serving as novel antibiotic drug [33].

Unfortunately, since in *C. sativa*, CBGA is promptly and directly converted to CBDA and THCA, leaving no CBGA pool available to form CBG, the CBG levels in plants are exceptionally low. In this context, it has been suggested that a possible strategy to increase the CBG yield from hemp biomass could consist in harvesting much earlier in the ripening phase of the plants before the other cannabinoids are formed and detract the CBGA from the cannabinoid pool [40]. On the other hand, having available reliable synthetic procedures to prepare natural PCs would consent the accessibility to considerable quantities of CBG, as well as of other microbiologically promising minor cannabinoids, unlikely provided naturally by *Cannabis* plants, thus allowing further studies finalized to the development of novel PCs-based antibiotics.

Particularly, since deprived of psychoactive effects, the non-psychotropic CBG, CBD, and CBC could represent promising compounds or template molecules for developing novel antibiotics with high therapeutic potential. Here, to give the reader a comprehensive background on the topic, we have first reviewed cannabinoids, in terms of classification, chemical structures, mode of action, main pharmacological properties, current applications, clinical applicability, and antimicrobial properties. Doing this, CBC, CBG and CBD, known to exclusively possess beneficial non-mind-altering pharmacological effects and reported to have potent antibacterial effects, have emerged as the most promising compounds for the development of new efficient antibacterial agents. So, with the aim to ease the synthetic production of high amounts of CBC, CBG and CBD for favoring further studies and for promoting the development of new antimicrobial agents, we have systematically reviewed all the synthetic pathways utilized for their preparation, reporting both reaction schemes and experimental details.

## 2. Phytocannabinoids (PCs), Endocannabinoids (ECs) and Synthetic Cannabinoids (SCs) 

### 2.1. Phytocannabinoids (PCs) and Endocannabinoids (ECs)

**Figure 2:**
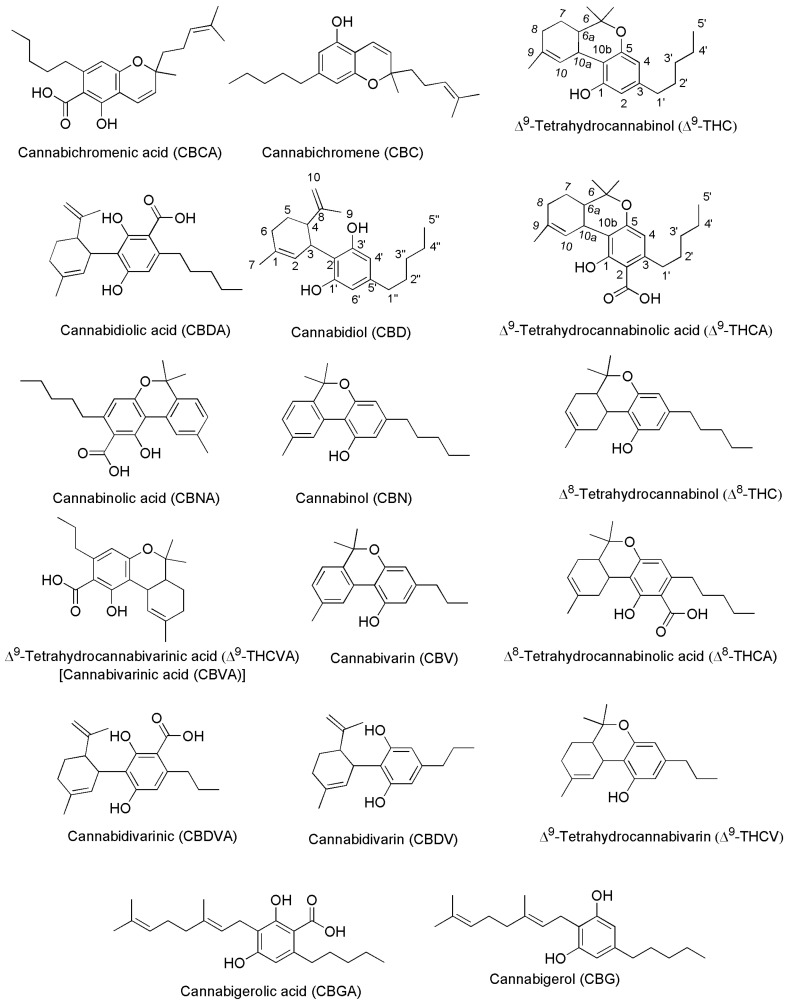
Chemical structure of the main PCs found in *C. sativa* acting on CB1 and/or CB2 receptors.

**Figure 3:**
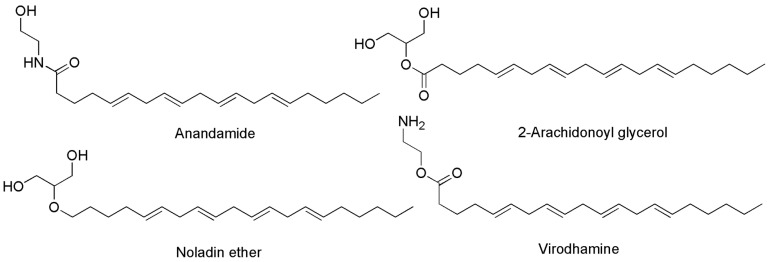
Chemical structure of the main ECs found in humans acting on CB1 and/or CB2 receptors.

Generally, the term ‘cannabinoids’ refers to a heterogeneous family of compounds that exhibit activity upon particular human cannabinoid receptors, namely CB1 and CB2 [41,42]. They encompass the natural compounds present in the *Cannabis* plants, lipid mediators called ECs naturally produced by human cells, as well as by all vertebrates on planet Earth, and the synthetic analogs of both groups designed by scientist, called SCs [42]. Natural cannabinoids from *Cannabis* are more specifically called PCs referring to their original plant source, differently from ECs which are produced from human cells [43,44]. PCs and ECs could include compounds structurally very different both between the two families and inside the same class, as shown in Figure 2 and Figure 3, which report the structure of the most relevant PCs and ECs, respectively. 

Both PCs and ECs exert their effects by interacting with CB1 and CB2 receptors, found throughout the human body, and whose locations have been listed in Table 2. We have constructed Table 2 using the valuable information contained in the relevant work by Fraguas-Sánchez et al. [45]. 

**Figure 4:**
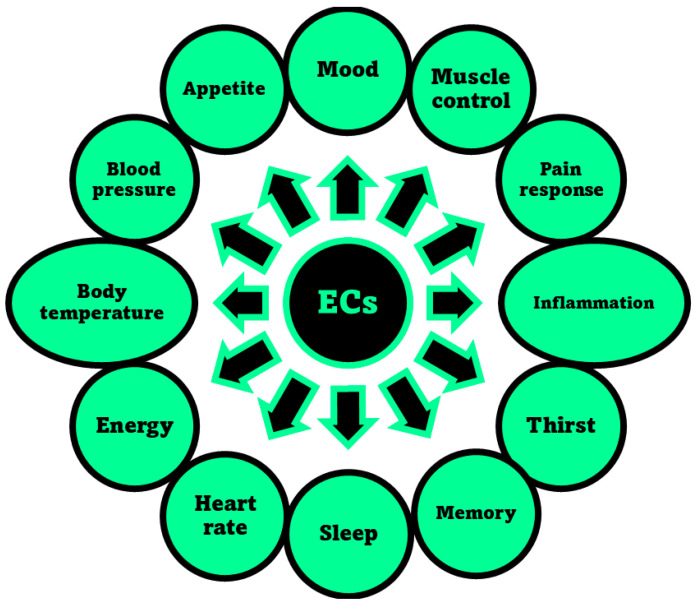
Aspects of humans’ life regulated by the ECS, through the interaction of ECs with receptors CB1 and/or CB2, as reported in the relevant review by Sharma et al. [52].

The activation of CB1 receptor or the concurrent activation of both receptors by ECs or PCs leads to both psychotropic, undesired effects and therapeutic outcomes. Exactly, while mind alteration, psychotropic effects, cardiovascular adverse events can occur, analgesic, sedative, antidepressant, anti-inflammatory, anti-anorexic, anti-emetic, anticancer and antibacterial desirable effects can also arise. As an example, the FDA-approved drug formulations containing the synthetic versions of Δ^9^-THC namely dronabinol (marketed as Marinol^®^ or Syndros^®^) or nabilone (marketed as Cesamet™), as well as the extracted THC (marketed as Sativex^®^), possess affinity for both CB1 and CB2 [48]. Clinically, they are primarily used to treat the chemotherapy-induced nausea, to enhance appetite in cachexic AIDS-patients, and to alleviate the spasticity and pain associated with multiple sclerosis [30]. Unfortunately, evidence of undesired psychotropic and cardiovascular adverse effects strongly limits the therapeutic efficacy of such medicines [49]. Otherwise, the selective activation of CB2 receptors, occurring for example by CBG or CBD, could provide therapeutic effects, such as immuno-modulatory properties, anti-inflammatory, anti-emetic, and anti-anorexic effects without exerting the psychotropic actions deriving from the CB1 activation. In addition, the potent analgesic effects, associated with the activation of CB2, could be helpful in alleviating chronic widespread musculoskeletal pain (CWP) disorders, such as fibromyalgia syndrome [50]. Also, the selective activation of CB2 receptors could enhance severe human diseases as osteoporosis, atherosclerosis, cancer, chronic liver injuries and neurodegeneration [48]. Collectively, CB1 and CB2 receptors together with ECs make part of the so-called EC system (ECS), which was discovered in the 1990′s by scientists researching cannabinoids, which includes also several enzymes involved in producing and recycling ECs. In humans, ECs are naturally produced by cells within the body in response to external factors, like pain or temperature. As shown in Figure 3, among other molecules, ECs include the well-known compounds 2-arachidonoylglycerol (2-AG) and anandamide (ANA), as well as the less-known ECs like virodhamine, and 2-arachidonoyl glycerol ether [51]. Particularly 2-AG and ANA activate both CB1 and CB2 receptors with affinity for CB1 higher than that for CB2 [48]. Collectively, the interaction between ECs and their corresponding receptors is pivotal in maintaining the body’s internal balance or homeostasis. ECs regulate some very important aspects of human health, as depicted in Figure 4.

**Figure 5:**
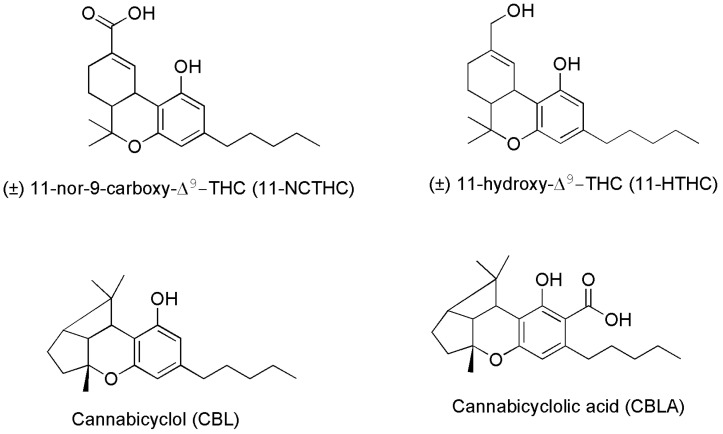
Chemical structure of two THC metabolites (11-NCTHC and 11-HTHC) and of two products deriving from CBCA degradation.

Researchers suggest that ECs deficiencies could cause many refractory health conditions, such as depression, arthritis, fibromyalgia, and Crohn’s disease that could ameliorate upon treatments with *Cannabis*, due to the activation by PCs of the same receptors activated in normal conditions by ECs. In fact, as reported above, PCs specifically produced by *Cannabis* plants and not by humans, when appropriately assumed can interact with CB1 and/or CB2 receptors triggering effects similar to those prompted by ECs, thus influencing the same aspects reported in Figure 4 and contributing to maintain or recover the body’s internal balance or homeostasis [41]. Anyway, if abused, the psychotropic and undesired side effects of psychoactive PCs, such as THC and CBN may overwhelm the benefits. Figure 5 shows the chemical structure of two metabolites which form in the human body after *Cannabis* consumption, of cannabicyclolic acid (CBLA), a degradative byproduct of cannabichromenic acid (CBCA), and of cannabicyclol (CBL) which is the product of decarboxylation of CBLA [53]. Particularly, CBL is a non-psychoactive cannabinoid, which could also derive by degradation of CBC through natural irradiation or under acid conditions [54]. 

Particularly, CBLA, like CBCA and CBC, is a minor cannabinoid found in low concentrations in the *Cannabis* plant. It is not produced by *Cannabis* directly, but it forms when CBCA degrades after exposure to ultraviolet (UV) light or heat. CBLA is an acidic cannabinoid, like THCA, CBCA and CBDA, which produces CBL, upon decarboxylation and release of CO_2_. CBLA is not considered intoxicating, is often deemed non-psychoactive or non-psychotropic, and curiously, it does not interact with receptors CB1 and CB2. There is little research into the effects and potential therapeutic uses of CBLA and CBL. Anyway, although more research is needed to confirm these attributes, some suggestions exist, that CBLA may have anti-inflammatory, antimicrobial, and antitumoral effects, due to its structural similarity to CBCA and CBN [53]. As for the metabolite 11-NCTHC, it is a no longer active secondary metabolite of THC, which forms in the body through the oxidation of the still psychoactive metabolite of THC, 11-HTHC, by liver enzymes [55].

#### Structural Differences between Psychotropic and Not-Psychotropic PCs

It has been reported that the n-pentyl chain at the C-(3) position (Figure 2) works and essential role in the activity of psychotropic THC derivatives and that modification in this side chain leads to critical changes in the affinity, selectivity and pharmaco-potency of these ligands relating to the CB1 and CB2 cannabinoid receptors. Generally, while a shorter alkyl chain reduces the affinity of the compound for the cannabinoid receptor, an increase in the number of carbon atoms (hexyl, heptyl, or octyl) leads to an increase affinity for the same cannabinoid receptor [56,57]. Additionally, a number of other transformations in the tricyclic core of the THC cannabinoid structure have been carried out [58]. Particularly, the pyran ring-opening generally causes in the achieved compound a relative reduction in the affinity to the CB1/CB2 cannabinoid receptors, and in the psycho activity. In this regard, the absence of the tricyclic core in CBC, CBD and CBG for CB2 receptors, could be responsible for the for their higher affinity for CB2 receptors dealing with beneficial pharmacological properties, thus not exerting psychotropic effects [59].

### 2.2. Synthetic Cannabinoids (SCs)

**Figure 6:**
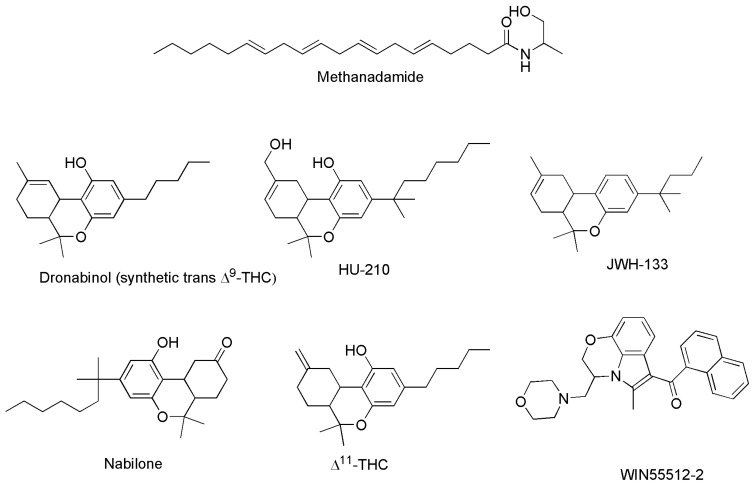
Structure of some SCs capable to act on CB1 and/or CB2 receptors.

The third group of cannabinoids consists of synthetic analogs of both ECs and PCs groups, appositely designed by scientists in the field, to enhance the benefits and therapeutic properties of ECs and PCs, while reducing the psychotropic and adverse effects. Among others, they include the compounds reported as examples in Figure 6 (chemical structures), and Table 3 (pharmacological properties and selectivity for receptors CB1 and CB2), which have demonstrated to be promising for treating severe humans’ chronic diseases including breast and prostate tumors, the unpleasant side-effects of chemotherapy, and chronic pain [42].

On the base of their affinity and selectivity for receptors CB1 and CB2, they can exert both therapeutic and psychotropic effects, or mainly one of the two. Table 3 summarizes the selectivity of some SCs for CB1 and CB2, and their therapeutic effects.

**Figure 7:**
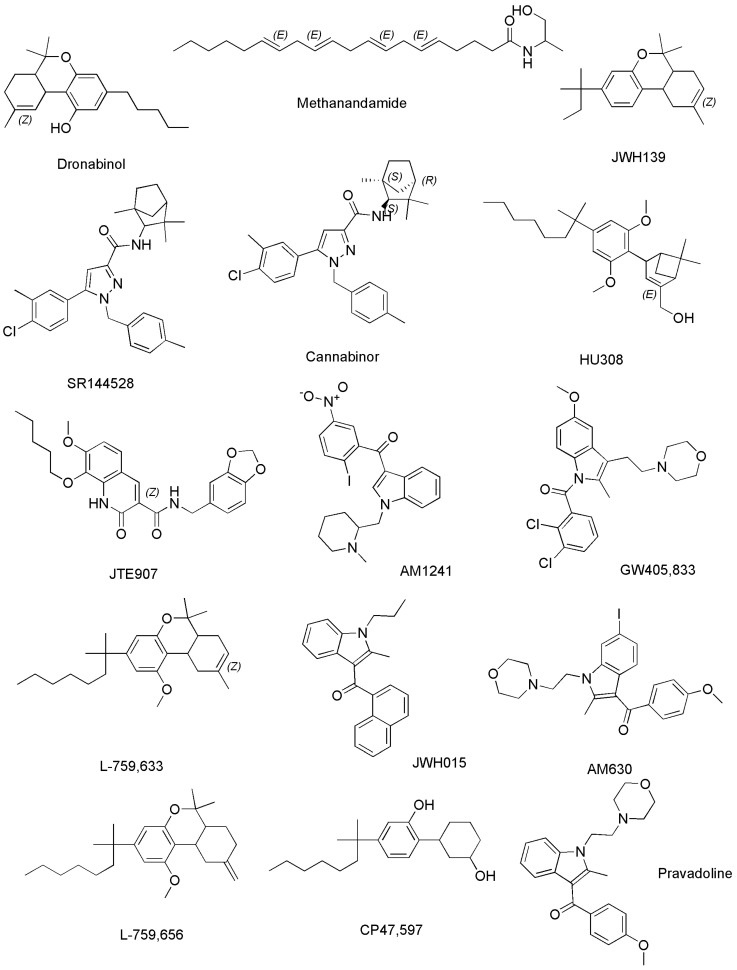
Structure of SCs capable to act on CB1 and/or CB2 reported in Table 3 and not previously shown in Figure 6.

The following Figure 7 shows the chemical structures of compounds in Table 3 not previously reported in Figure 6. 

Methanandamide (AM-356) is a synthetically constructed stable chiral analog of anandamide. AM-356 acts on the cannabinoid receptors, and specifically on CB1-type receptors in the CNS found in mammals, fish, and certain invertebrates (e.g., Hydra), thus resulting also a psychoactive compound [68]. HU-210, as well as other SCs including L-759,656, HU-308, L-759,633, L-768,242 etc. are potent analgesic and anti-inflammatory compounds with many of the same effects as natural THC [44]. WIN 55,212-2 is an organic heterotricyclic SC. Particularly, it is the 5-methyl-3-(morpholin-4-ylmethyl)-2,3-dihydro [1,4]oxazine [2,3,4-hi]indole substituted at position 6 by a 1-naphthylcarbonyl group. It has a role as an analgesic, and neuroprotective agent, as well as an apoptosis inhibitor [69].

JWH-133 is a Δ^9^-tetrahydrocannabinol lacking the hydroxy group and having a 1,1-dimethylbutyl group at position 3 in place of the pentyl group. It acts as potent and highly selective CB2 receptor agonist, thus exerting antineoplastic effects, and working as a vasodilator and an anti-inflammatory agent, as an apoptosis inhibitor, as well as an analgesic molecule [70].

Δ^11^-THC, also known as exo-tetrahydrocannabinol, is a synthetic isomer of tetrahydrocannabinol, developed in the 1970s. It can be synthesized from Δ^8^-THC by several different routes, and only the (6aR, 10aR) enantiomer is known. In animal studies in mice, it was found to exert the same effect of Δ^9^-THC with around 1/4 its potency. It has been identified as a component of “vaping liquids” sold for use in humans [71].

### 2.3. Cannabinoids Clinically Approved

Collectively, PCs and the several developed SCs have proved to be useful in the treatment of chemotherapy side effects such as nausea, vomiting, pain, weight loss, and lack of appetite [30], but only few drugs based only on THC and CBD have been approved so far in some countries, as palliative agents in anticancer treatments. Dronabinol, the synthetic analogous of Δ^9^-THC and nabilone, a SC similar to Δ^9^-THC, are currently approved in Canada, United States, and several countries in Europe to treat nausea and vomiting associated with chemotherapeutic treatments [72]. An oromucosal spray containing a mixture 1:1 of Δ^9^-THC and CBD marketed as Sativex^®^ is approved in Europe and Canada for the treatment of spasticity associated with multiple sclerosis (MS), while in Canada Sativex is applied also as an adjunctive analgesic for the treatment of pain in patients with advanced cancer and MS [73,74]. An oral solution of CBD, marketed as Epidiolex^®^ is an US FDA-approved prescription that is used in association with clobazam to treat refractory epilepsy due to Lennox–Gastaut or Dravet syndrome [75,76]. Finally, we signalize the case of Rimonabant (or SR141716), which was marketed as Acomplia^®^. It is an inverse agonist for the CB1 receptor, capable to reduce the appetite, and was clinically applied as an anorectic anti-obesity drug. It was withdrawn from the market in 2009, after a long dispute between the European Medicines Agency (EMA) and the Cochrane Collaboration, because it increased the risk of psychiatric problems and suicide [77].

## 3. Phytocannibinoids: Polyfunctional Molecules Promising to Develop Novel Antibiotics

### 3.1. Not Only THC and CBD

Wehave likely heard that *C. sativa* provides THC, having high medicinal value, but also mind-altering effects (psychotropic) and cannabidiol (CBD), which is praised for its medicinal benefits without being psychoactive [41]. Anyway, while these are the most well-known and abundant PCs, as mentioned above, there are a plethora of other cannabinoids produced by the *Cannabis* plants.

Particularly, *C. sativa* produces about 1600 chemical substances, 500 bioactive compounds, including approximately 180 cannabinoids (113–125 isolated), many terpenes, and flavonoids which could be promising as novel therapeutic agents [40,78].

**Figure 8:**
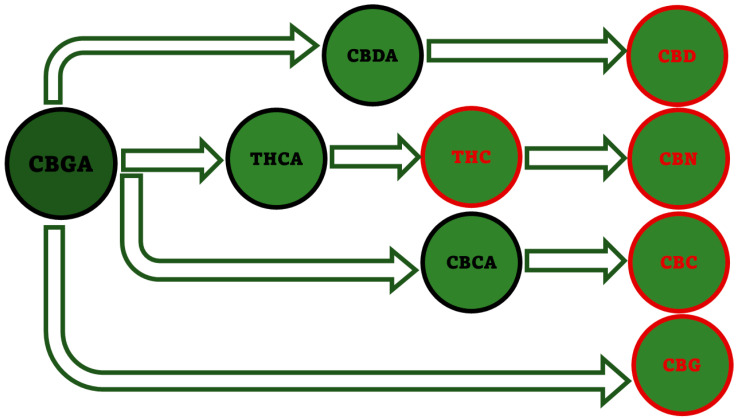
Biosynthetic pathway starting from CBGA and leading to the five major PCs. This image has been created by the authors exploiting information available online at https://www.openaccessgovernment.org/cbg-the-mother-of-all-cannabinoids-with-broad-antibacterial-activity/95824/ (accessed on 3 May 2023).

More generally, the known different cannabinoids, are naturally present not only in *Cannabis*, but also in various other plants or in burned cannabis resin. Cannabinoids are found in certain species of flowering plants (Rhododendron), some species of liverworts, an ancient fern-like plant, as well as in certain types of fungi (myco-cannabinoids). Among PCs, cannabigerolic acid (CBGA) which is the direct precursor of the decarboxylated CBG, can be considered as the “mother of all major cannabinoids”, according to the biosynthetic pathway in Figure 8 [40].

The most common misunderstanding about *Cannabis* consists in thinking that the plant produces THC, as well as the other major activated cannabinoids (red compounds in Figure 8), while it actually generates their acidic forms (raw cannabis), such as CBGA, THCA, CBDA, CBCA, which are converted in the decarboxylated forms (CBG, THC, CBD, CBC) only once the flower is heated. Actually, very little activated cannabinoids are found in fresh *Cannabis* flower, while most are in the acidic form and get decarboxylated into THC, CBD, CBC or CBG upon smoking or plant ageing [41]. Anyway, despite being most dismissed as inactive, because it is not absorbed in the brain, also acids cannabinoids may actually offer several therapeutic potentials, including antimicrobial activity.

### 3.2. Much More beyond the Psychotropic Effect of THC

**Figure 9:**
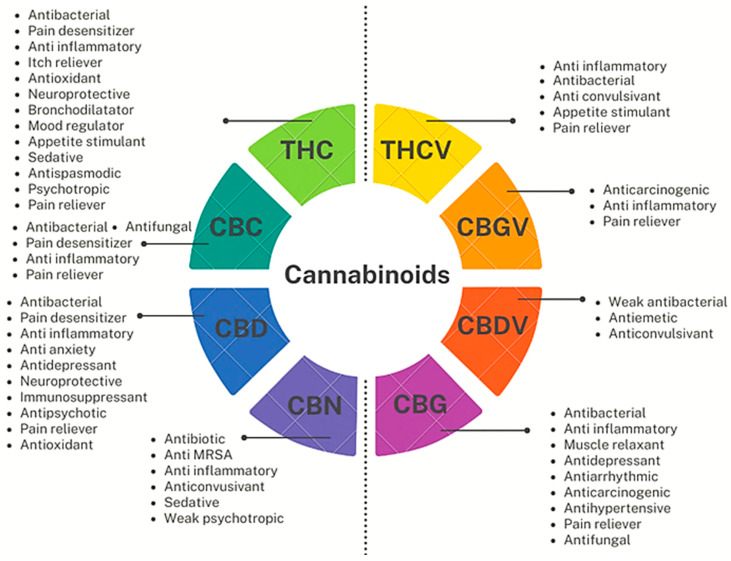
Pharmacological properties of some relevant major and minor PCs. Figure 9 has been originally created by the authors using information found online [41] and using Flipsnack, a free dowlodable application to produce PowerPoint professional designes, available online at https://app.flipsnack.com/editor/7uld8l9xu3 (accessed on 14 June 2023).

Figure 9 shows the several pharmacologic activities of some relevant natural cannabinoids (PCs), including also some minor cannabinoids of the varin (V) family.

From a structural point of view, CBDV, THCV and CBGV reported in Figure 9 and CBCV (not reported), belonging to the varins (Vs), have two fewer carbon atoms in the alkyl chain with respect to the correspondent better-known cannabinoids CBD, THC, CBG and CBC. This shorter carbon chain strongly affects their pharmacologic activity, thus being promising compounds in managing weight loss, diabetes, cholesterol problems, autism, seizures, and more [79]. Particularly, THCV, although very structurally similar to THC has a totally different effects profile. The slight alteration in its chemical structure implies that, unlike THC, it acts as an antagonist to the receptor CB1 rather than an activator, thus not producing relaxing, euphoric, and energizing outcomes, but exerting antianxiety, anticonvulsant, appetite suppressant and anti-inflammatory effects [41]. THCV could be a weight-loss aid, by reducing appetite and boosting metabolism, and could be helpful in the treatment diabetes by controlling the blood sugar levels and the insulin production. Also, THCV may help promoting new bone cell growth, preventing weakening bones, and can even act as a neuroprotectant in conditions like Parkinson’s disease [80].

**Figure 10:**
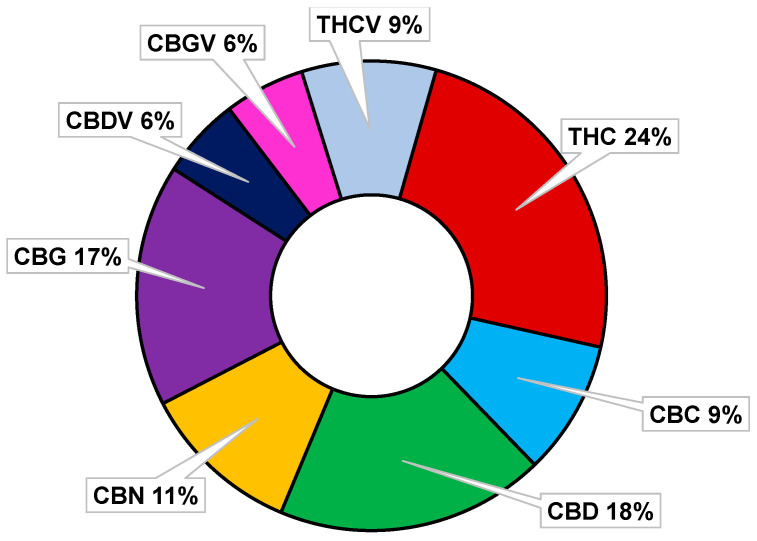
Percentages of pharmacological properties possessed by some major and minor cannabinoids.

Collectively, as observable both in Figure 9 and in the following Figure 10, all the reported cannabinoids, like many other phytochemicals, are multifunctional compounds owing several pharmacological activities, being THC, the cannabinoid possessing the highest number of effects on human body, followed by CBD and CBG.

Interestingly, only THC and CBN possess intoxicant effects (psychotropic actions), while all other PCs in Figure 9 and Figure 10, except for CBGV, have demonstrated to possess antimicrobial properties, and especially antibacterial effects on MDR strains of Gram-positive species, thus being promising molecules or template compounds to develop novel antibiotics. The molecular mechanisms at the base of the antibacterial activity of PCs have yet to be fully unveiled. Anyway, the effects of structural modifications on the bactericidal effects of the more studied cannabinoids (CBD, CBC, CGB, THC, and CBN) have been recently investigated by Scott et al. [81]. All the considered cannabinoids demonstrated potent activity against a variety of MRSA strains, with MIC values in the range 0.5–2 µg/mL. Methylation and acetylation of the phenolic hydroxyls, esterification of the carboxylic group, as well as the introduction of a second prenyl group were detrimental to the cannabinoids’ antibacterial activity. The antibacterial effects were maintained regardless the type of prenyl moiety, its relative position compared to the n-pentyl moiety (abnormal cannabinoids), and the carboxylation of the resorcinol moiety (pre-cannabinoids). Collectively, structural modification of the terpenoid moiety do not affect the antibacterial effects of PCs, suggesting that these residues serve mainly as modulators of lipid affinity, while the addition of further prenyl moiety may result in poorer aqueous solubility, leading to a loss of antibacterial activity [81].

### 3.3. Antimicrobial Cannabinoids

**Figure 11:**
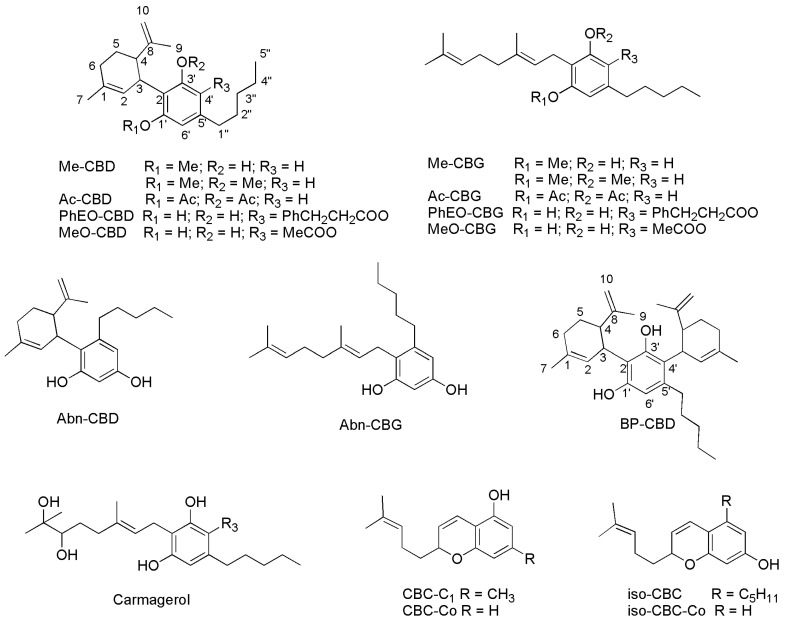
SCs reported in Table 4 whose chemical structure was not reported previously. Abn-CBD and Abn-CBG are defined as *orto*-isomers of *para*-derivatives CBD and CBG, being the *orto*- and *para*-positions those reciprocals to the pentyl chain.

Table 4 reports the most relevant PCs, both in the acid forms and in the decarboxylated ones, ECs, and SCs (mainly prepared by scientist to study the structure-activity relationships (SARs), which were in the past or recently assayed as antimicrobial agents. Figure 11 shows the chemical structure of SCs present in Table 4 not showed previously, while the subsequent Table 5 reports the antimicrobial properties of compounds listed in Table 4, expressed as minimum inhibitory concentrations (MICs), as well as the target pathogens.

Since except for compounds Δ^11^-THC, Abn-CBD, Abn-CBG and CBC derivatives, the other SCs reported in Table 4 and Figure 11 demonstrated insignificant activity against the tested pathogens reported in Table 5 (MICs > 100 µg/mL), they were no longer reported in Table 5.

The first data we have found concerning the possible antibacterial activity of PCs were reported by Van Klingeren and Ham in the year 1976 [82]. The authors tested both THC and CBD on *S. aureus* and some isolates of *Streptococcus* genus, finding MICs in the range 1–5 µg/mL for both compounds against both species [82]. A weak antifungal activity was reported in the past (years 1981, 1982) only for CBC, some its synthetic derivatives [87] and for CBG [89], but no recent reports is present in the literature, thus demonstrating the poor interest of scientists, probably due to the scarce effects. On the contrary, in the year 1981, Turner found good antimicrobial activity for CBC against *Bacillus subtilis* (MIC = 0.4 µg/mL) and *S. aureus* (1.6 µg/mL), and for ICBC against *B. subtilis* (MIC = 0.8 µg/mL) [87]. ∆^9^- THC, CBD, CBG, CBC, and CBN were assayed for their antimicrobial properties by Appendino et al. on the MDR *S. aureus* SA-1199B strain, which showed a high level of resistance to certain fluoroquinolones, on EMRSA isolates, which are the major epidemic methicillin-resistant *S. aureus* strains occurring in U.K. hospitals, on a macrolide-resistant strain (RN4220), on a tetracycline-resistant line (XU212), and on a standard laboratory *S. aureus* strain (ATCC25923) [83]. All compounds showed potent antibacterial activity, with MIC values in the 0.5–2 µg/mL range. Interestingly, also the acidic precursors of CBD, CBG, and THC (compounds CBDA, CBGA and THCA) maintained the activity substantially [83].

Since given their non-psychotropic profiles, CBD and CBG can be considered especially promising, Appendino et al. performed structure-activity studies. Among the various synthetic derivatives, only the synthetic abnormal cannabinoids Abn-CBD and abn-CBG, although slightly less potent than CBD and CBG, showed antibacterial activity comparable to that of their corresponding natural products [83].

According to recent reports, 18 cannabinoids including CBC, CBD, CBG, CBN, Δ^9^-THC and their carboxylic precursors (pre-cannabinoids CBCA, CBDA, CBGA, Δ^9^-THCA), CBDV, THCV and their precursors (CBDVA, THCVA), Δ^8^-THC, CBL, 11-NCTHC, 11-HTHC and Δ^11^-THC were tested against MRSA USA300, a highly virulent and prevalent community-associated MRSA, by Farha et al. [33]. Susceptibility tests were conducted according to the Clinical and Laboratory Standards Institute (CLSI) protocol [91].

CBG, CBD, CBN, CBCA, Δ^9^-THC, Δ^8^-THC, and Δ^11^-THC were potent antibiotics with MICs = 2 µg/mL. A moderate loss of potency was observed for their acidic precursors such as CBDA, CBGA and THCAA. THCV and CBDV were less active, displaying MICs = 4 and 8 µg/mL, respectively. It is well-known, that biofilm formation by MRSA, typically on necrotic tissues and medical devices, represents an important virulence factor influencing the persistence of MRSA and is typically associated with increased resistance to antimicrobial compounds. In this regard, Farha et al. investigated also the capability of the above-mentioned cannabinoids to inhibit the formation of biofilms by MRSA [33]. According to the results reported, the tested cannabinoids except for varins, clearly repressed MRSA biofilm formation, with CBG exhibiting the most potent antibiofilm activity. Indeed, at concentration of 0.5 µg/mL (1/4 MIC), CBG inhibited biofilm formation by ∼50% [33].

When its effect was evaluated on preformed biofilms by determining its minimal biofilm eradication concentration (MBEC), CBG eradicated preformed biofilms of MRSA USA300 at concentration of 4 µg/mL. Additionally, on MRSA persister cells, which are a nongrowing, dormant cells subpopulations, which exhibit high levels of tolerance to antibiotics, and are responsible of chronic and relapsing *S. aureus* infections, such as osteomyelitis and endocarditis, the tested cannabinoids showed antipersisters activity which correlated with MIC values [33]. Again, CBG was the most potent cannabinoid against the persisters. In time-kill experiments, while the β-lactam oxacillin at 160 µg/mL (5 × MIC) did not show any activity, CBG killed persisters in a concentration-dependent manner starting at 5 µg/mL, and rapidly eradicated a population of ∼10^8^ CFU/mL MRSA persisters within 30 min of treatment.

As expected, the two most common human metabolites of THC, (±)11-NCTHC and (±) 11-HTHC, as well as CBL were inactive at the highest concentrations screened (MIC > 32 µg/mL). Unfortunately, in the study by Farha et al., none of these analogous displayed bactericidal effects against *E. coli* [33,78]. However, CBG was found to be effective also against Gram-negative bacteria when associated to polymyxin B or the less nephrotoxic polymyxin B nonapeptide [33,78] and acted as a sensitizing agent in combination with various antibiotics [92].

Concerning the association with polymyxin B, it was proposed that polymyxins permeabilize the outer membrane of Gram-negative pathogens, unassailable by CBG, thus enabling CBG to reach and damage the inner membrane [33,78]. Additionally, when *E. coli* VCS257 was treated with CBD in combination with erythromycin, vancomycin, rifampicin, kanamycin or colistin, an enhanced antimicrobial effect was observed [92]. In the year 2020, Martinenghi et al., tested CBDA and CBD against *S. aureus* and *S. epidermidis* finding very low MIC for CBD (MIC = 1 µg/mL and 2 µg/mL, respectively), and MICs twice as high for CBDA, while CBDA displayed MIC = 4 µg/mL against MRSA USA300 [80]. In the same year, Wassmann et. al. reported for CBD, MICs = 4, 4, 4, and 8 µg/mL against MRSA USA300, MRSE, *L. monocytogenes* and *E. faecalis* respectively [86]. One year later, MICs in the range 1–5 µg/mL were reported for CBG, against some species of *Streptococcus* genus [88].

Feldman and colleagues tested ANA and AraS, which are the main ECs found in humans against MRSA [90]. While they resulted completely inactive towards planktonic cells (MICs > 256 µg/mL), they demonstrated appreciable activity against MRSA biofilm, by reducing the biofilm formation by 51–54% (ANA) and 33–61% (AraS) at concentration 64 µg/mL [90]. Very recently, the antibacterial and antioxidant properties of CBD and its homologue, 8,9-dihydrocannabidiol (H2CBD), were also examined by Wu et al. against *S. aureus* and *E. coli* with excellent results against both species with both compounds [85]. On these findings, *C. sativa* and its non-psychotropic cannabinoids, represent an interesting source of novel antibacterial agents which could help in addressing the problem of multidrug resistance in MRSA and other pathogenic bacteria.

## 4. Production of Phytocannabinoids: From Biosynthesis to Synthetic Procedures

### 4.1. Biosynthesis of Non-Psychotropic Cannabinoids (CBC, CBG and CBD)

**Scheme 1:**
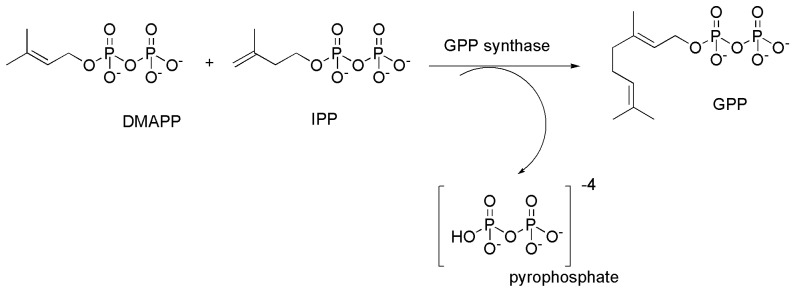
Synthesis of geranyl pyrophosphate (GPP) from dimethylallyl pyrophosphate (DMAPP) and isopentenyl pyrophosphate (IPP) catalyzed by geranyl pyrophosphate synthase.

**Scheme 2:**
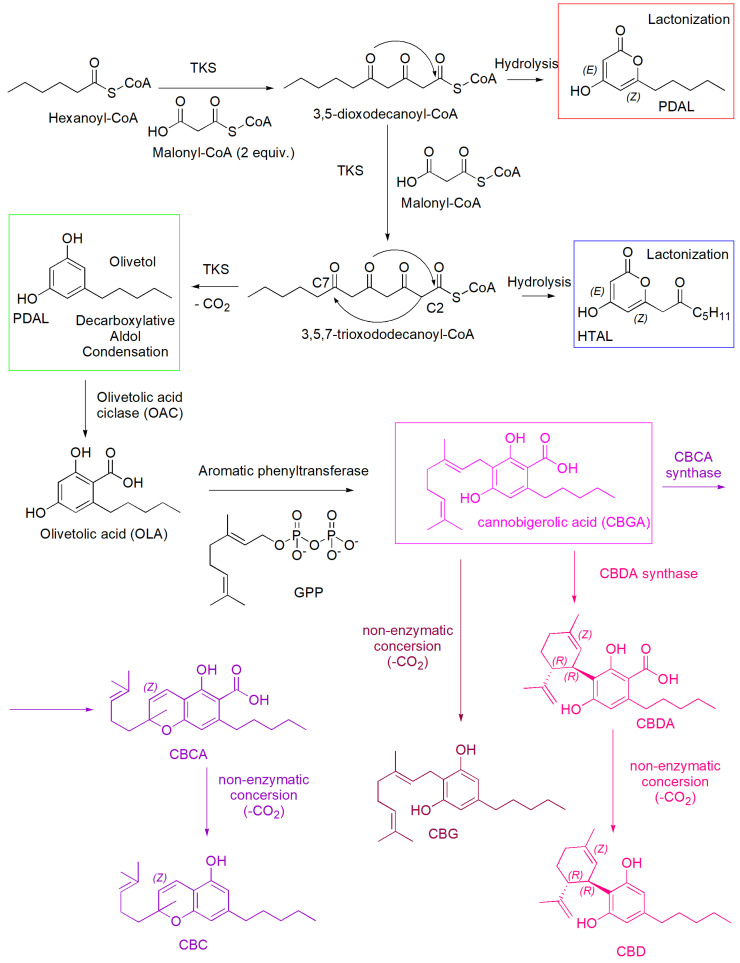
Biosynthesis of non-psychotropic cannabinoids CBG (amaranth route), CBD (pink route) and CBC (light purple route), including the formation of the three by-products pentyl diacetic lactone (PDAL), hexanoyl triacetic acid lactone (HTAL), and olivetol.

The following Scheme 1 and Scheme 2 show the biosynthetic path to form geraniol pyrophosphate (GPP) and leading to CBG, CBC and CBD starting from CBGA, respectively [13]. As reported in the previous Figure 8, in *C. Sativa*, from CBGA, THCA is also produced, which provides THC upon decarboxylation, which in turn gives CBN, upon oxidation and aromatization. Although they have demonstrated interesting antibacterial effects, THCA, THC and CBN were not considered in this section because they possess from weak to strong psychotropic effects, which limit their possible therapeutic use [35]. On the contrary CBC, CBG and CBD, not exerting psychotropic actions have higher therapeutic potentials thus being more suitable to develop novel antibiotics [17].

Briefly, the tetraketide synthase (TKS) catalyzed sequential condensation of hexanoyl-CoA with three molecules of malonyl-CoA yields 3,5,7-trioxododecaneoyl-CoA. By olivetolic acid cyclase (OAC), this compound cyclizes and aromatizes, through the loss of Coenzyme A, providing olivetolic acid (OLA) [13]. During these first steps, by hydrolytic processes and lactonization the side-products pentyl diacetic lactone (PDAL) (red square) and hexanoyl triacetic acid lactone (HTAL) (blue square) are also produced, while upon a decarboxylation TKS catalyzed, olivetol is formed (green square). Then, aromatic prenyltransferase inserts the prenyl group at the highly nucleophilic 2-resorcinol position to provide cannabigerolic acid (CBGA) [13]. Then, while CBGA provides CBG upon a non-enzymatic loss of CO_2_, through reactions catalyzed by the opportune synthases, it provides the cannabinolic acids CBDA, and CBCA, which in turn provide the de-carboxylate (−)-CBD and CBC, by non-enzymatic decarboxylation [13].

### 4.2. Synthetic Procedures to Prepare Non-Psychotropic Cannabinoids CBC, CBG and (−)-CBD

The synthetic procedures for synthesizing CBC, CBD and CBG reported in the following sections have been found upon a survey carried out using SciFinder^n^ data base (Chemical Abstracts Service (CAS)), available online at https://scifinder-n.cas.org/ (accessed on 3 May 2023). The research was performed using the CAS registry number of the compounds, and all the synthesis reported so far have been described. Patents have been excluded.

#### 4.2.1. Syntheses of CBC

**Scheme 3:**
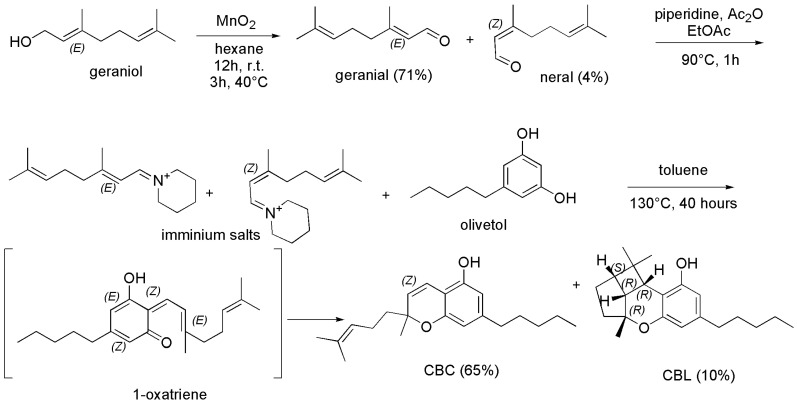
Synthetic procedure to achieve CBC [93]. Ac_2_O = acetic anhydride; EtOAc = ethyl acetate.

The most recent synthetic procedures for preparing CBC were reported in the year 2021. Particularly, Seccamani et al. [93], who reproduced synthetic procedures previously reported [94,95,96,97], synthesized CBC starting from *E*-geraniol according to Scheme 3.

**Scheme 4:**
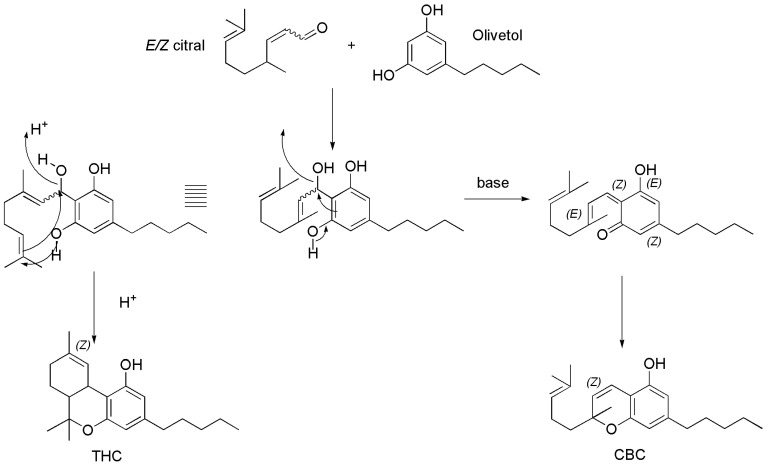
Synthetic procedure to achieve either CBC in basic conditions or THC in acidic ones [98].

Briefly, *E*-geraniol dissolved in dry hexane was treated with manganese dioxide (MnO_2_) under magnetic stirring at room temperature for about 12 h and then heated at 40 °C for further 3 h, to provide citral as crude product, which was purified by silica gel chromatographic column obtaining the purified aldehydes (with a yield of 75%) as an *E/Z* mixture (*E/Z* 95/5, GC-MS) of geranial (*E*-citral, 71%) and neral (*Z*-citral, 4%). Subsequently, the prepared mixture was treated with Ac_2_O in EtOAc in the presence of piperidine and heated at 90 °C for 1 h, to give an iminium salt, which was added with a solution of olivetol in toluene and stirred at 130 °C for 40 h. Upon an oxa-annulation consisting of a Knoevenagel reaction providing the 1-oxatriene intermediate, followed by an oxa-electrocyclization, the crude CBC was obtained, which was then purified by chromatographic column. Pure CBC (yield 65%) and cannabicyclol (CBL, yield 10%) were finally isolated. Interestingly, Luo et al. [94], and Yeom et al. [96], who previously employed this procedure starting from a mixture of *E/Z*-citral, and from only geranial (*E*-citral) respectively, achieved CBC with a yield lower than Seccamani (50% vs. 65%). Similar procedures starting from *E/Z*-citral and olivetol were proposed in the same year (2021), by Schafroth, et al. [98] and by Anderson et al. [99], achieving CBC with yields of 50% and 35%, respectively. Particularly, the group of Schafroth evidenced that acidic or basic conditions were determinant to redirect the reaction towards the formation of CBC rather than towards that of Δ^9^-THC (Scheme 4).

**Scheme 5:**
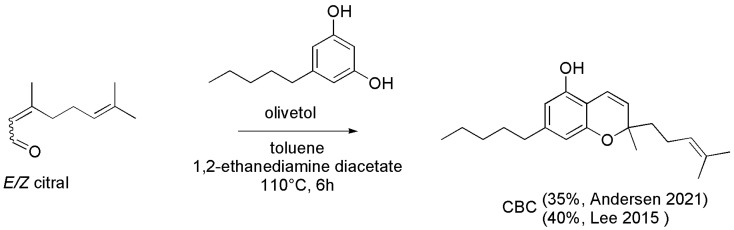
Synthesis of CBC [99,100].

Differently and more specifically, Anderson et al. reacted olivetol and *E/Z*-citral in toluene using ethylenediamine diacetate as catalyst and heating the solution at reflux for 6 h (Scheme 5), as it was reported previously by Lee et al. [100], who prepared CBC with similar yield (40% vs. 35%).

**Scheme 6:**
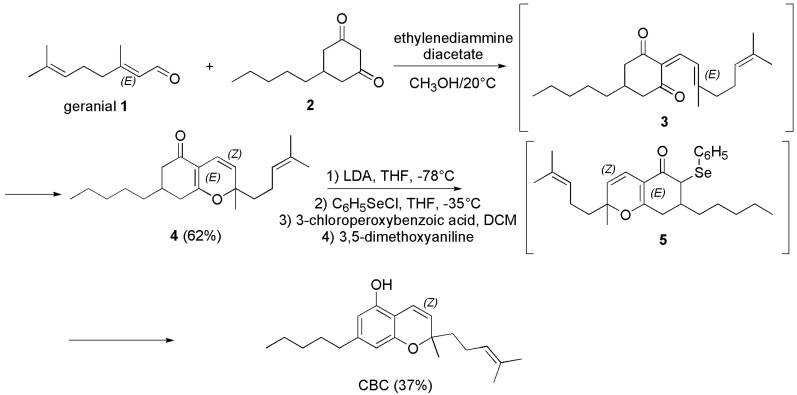
Synthesis of CBC [101].

The use of ethylenediamine diacetate as catalyst had been reported in the past by Tietze et al. in the year 1982 [101], who achieved CBC in similar yield (37%) according to a different path (Scheme 6).

Briefly, geranial (**1**) was reacted with 5-pentyl-1,3-cyclohexandion (**2**) in methanol (CH_3_OH) with catalytic amounts of ethylenediamine diacetate at 20 °C, achieving the intermediate **3**, which cyclized to the crude compound **4**. A chromatographic column was necessary to purify **4**, which was isolated in 62% yield. Compound **4** was treated with lithium di-isopropyl-amide (LDA) in tetrahydrofuran (THF) at −78 °C and phenyl-selenenyl chloride (C_6_H_5_SeCl), to afford the selenide intermediate **5**, which upon oxidation with 3-choloroperoxybenzoic acid in dichloromethane (DCM) followed by reaction with dimethoxy aniline, provided CBC in 37% yield.

**Scheme 7:**
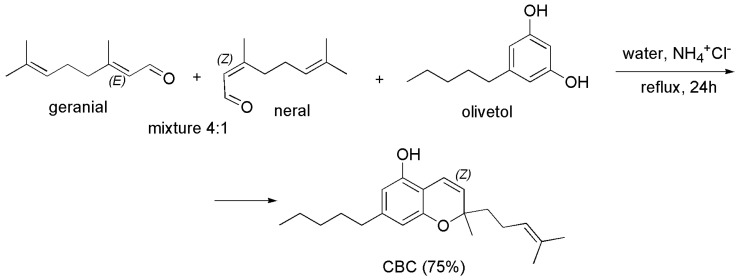
Synthesis of CBC [103].

An analogous procedure, using *t*-butylamine in place of ethylenediamine diacetate, and at reflux time of 9 h in place of 6, had been reported in the years 1978 and 1982 by Elsohly et al. [89,102]. CBC was achieved in high yield (>60%), upon purification carried out reducing the unreacted citral with NaBH_4_. In the 2008, the same reaction was exploited in their study by Appendino et al. [83]. Interestingly, CBC was prepared in very high yield (75%) by Quilez del Moral et al. [103], by a biomimetic green approach using water as solvent and ammonium chloride as catalyst, according to Scheme 7.

Particularly, working on a milligrams scale, the authors started from the commercial citral, as a mixture 4/1 of geranial (*E*-citral) and neral (*Z*-citral), which was reacted with olivetol in water using ammonium chloride (NH_4_^+^Cl^−^) as catalyst for 24 h at reflux. The obtained crude product was purified by chromatographic column, thus isolating CBC in 75% yield. The procedure is interesting, because depending on the use of a surfactant as sodium dodecyl sulfate (SDS) or that of NH_4_^+^Cl^−^ as catalyst, it was possible for the author to achieve an “in water” reaction thus obtaining ortho-THC as main product (CBC 45% yield), or an “on water” reaction achieving CBC as major compound (75% yield).

**Scheme 8:**
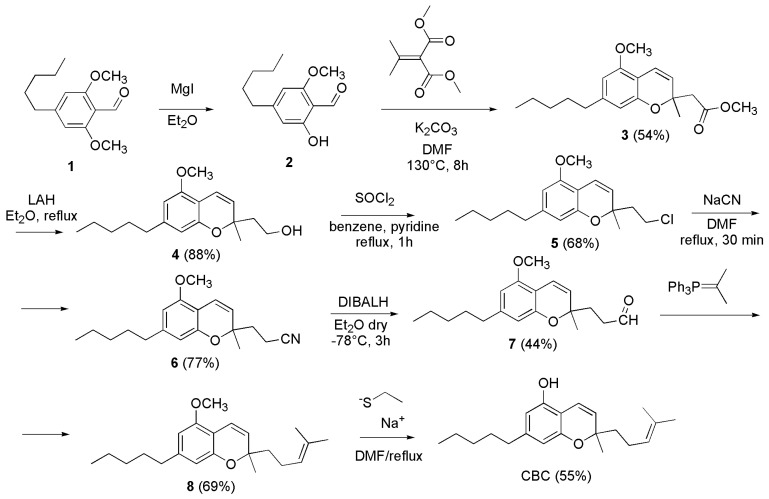
Synthesis of CBC [104].

In the past (year 1995), a multi-step synthesis for CBC was reported by Yamaguchi et al. (Scheme 8) [104].

Briefly, the 2-hydroxy-6-methoxy-4-pentylbenzaldehyde (**2**) was prepared demethylating **1** with magnesium iodide etherate. Then, **2** was cyclized to **3** using dimethyl isopropylidenemalonate and K_2_CO_3_ in dimethylformamide (DMF) at 130 °C for 8 h, obtaining the chromene-2-acetate derivative **3** in 54% yield. Subsequently, **3** was converted to the aldehyde **7**, by reduction with lithium aluminum hydride (LAH), chlorination with SOCl_2_, cyanation with NaCN and final reduction with diisobutylaluminium hydride (DIBALH). A Wittig reaction of **7** with isopropylidenetriphenylphosphorane provided *O*-methylcannabichromene (**8**) which was demethylated to CBC (yield 55%) by treatment with sodium ethanethiolate in refluxing DMF.

#### 4.2.2. Synthesis of CBG

**Scheme 9:**
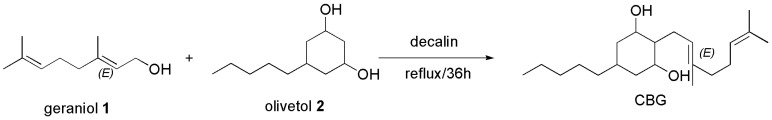
Synthesis of CBG [105].

The oldest synthetic procedure to prepare CBG we found, not reporting the reaction yield, was described by Gaoni et al. in the year 1964 [105]. The authors synthesized CBG by boiling geraniol (**1**) with olivetol (**2**), in decalin for 36 h (Scheme 9).

**Scheme 10:**
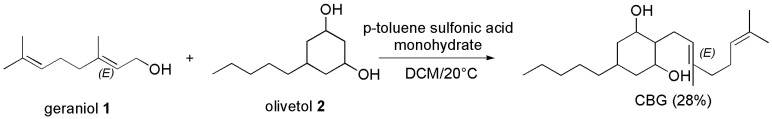
Synthesis of CBG [102].

Similarly, CBG was prepared by the condensation of geraniol and olivetol, using DCM in the presence of *p*-toluenesulfonic acid (PTSA) at 20° to achieve CBG as crystalline material in 52% yield, by Mechoulam et al. according to Scheme 10 [106].

Starting from the same materials (geraniol and olivetol) and using DCM as solvent and PTSA monohydrate as catalyst, Farha et al. [33] prepared CBG like Mechoulam et al. [106] with the same yield, reproducing the procedure previously reported by Taura et al. [107].

The reaction was stirred at room temperature in the dark for 12 h, then added with aqueous saturated NaHCO_3_. After evaporation of the separated organic phase, a crude residue was obtained, which was purified via flash column chromatography on silica gel, providing pure CBG as an off-white powder in 28% yield.

**Scheme 11:**
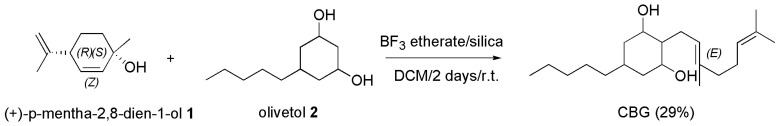
Synthesis of CBG [108]. Silica = Si_2_O_3_.

In 1985, it was reported that when BF_3_-etherate on silica was used as condensing agent in the reaction of (+)-*p*-mentha-2,8-dien-l-ol (**1**) with olivetol (**2**), CBG could be obtained as the major product, in 29% yield (Scheme 11) [108].

Particularly, BF_3_-etherate was added under nitrogen to a stirred suspension of silica in dry DCM, added with *Z*-(+)*-p*-mentha-2,8-dien-l-ol and olivetol dissolved in DCM and stirred at room temperature for 2 days. After having quenched the reaction with an aqueous solution of sodium bicarbonate followed by extraction with diethyl ether, CBG was achieved in 29% yield.

Later, the same authors used the above-reported procedure starting from geraniol and olivetol, as in Scheme 9 and Scheme 10, achieving CBG in 29% yield, as well [109,110].

A chemoenzymatic synthesis of CBG was reported by Kumano et al. in the year 2008 [111], which we did not discussed in the present work, because out of our scope aiming at describing only processes totally synthetic.

In the year 2020, Jentsch et al. reported the optimized synthesis of three phenolic natural products with unprecedented efficiency, using a new alumina-promoted regioselective aromatic allylation reaction [112]. As for CBG, it was prepared in one step from the inexpensive olivetol and geraniol, as in the reactions implemented previously by Farha et al. [33], Mechoulam et al. [106] and Taura et al. [107], but using different reagents and conditions, and achieving CBG in higher yield.

Briefly, to a solution of geraniol and olivetol in dichloroethane (DCE), acidic alumina (Al_2_O_3_) was added, and the heterogeneous mixture was stirred at reflux temperature for 6 h. After filtration of the alumina and the removal of the organic solvent, CBG was achieved as a yellow oil, that was purified via chromatography, thus obtaining pure CBG in 62% yield.

**Scheme 12:**
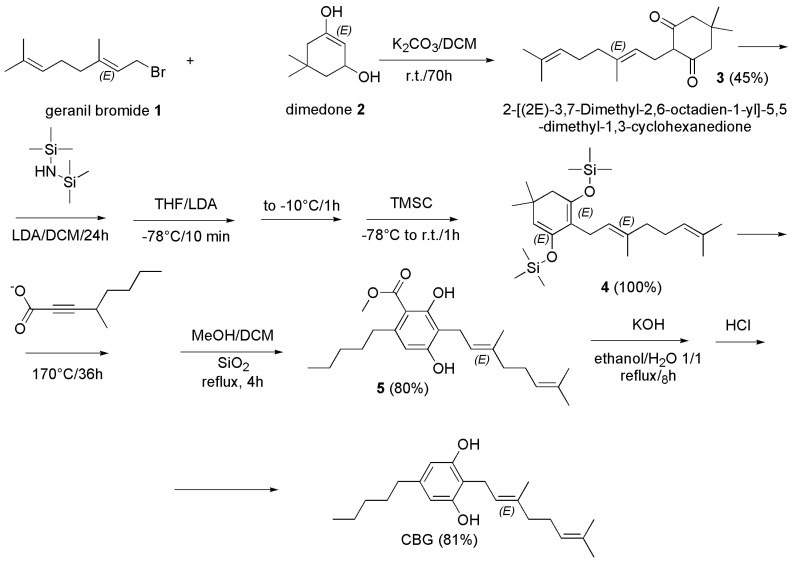
Synthesis of CBG [113].

Like for CBC, the most recent synthetic procedures for preparing CBG have been reported in the year 2021. The group of Curtis et al. reported a multistep procedure based on a tandem Diels–Alder/retro-Diels–Alder cycloaddition which allowed to achieve CBG in very high yield (81%) (Scheme 12) [113].

**Scheme 13:**
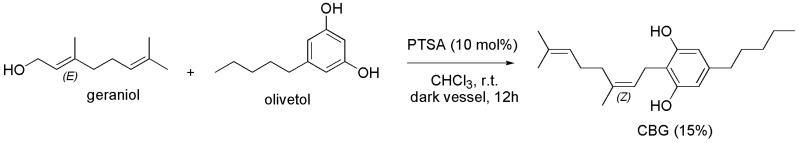
Synthesis of CBG [93].

The group of Seccamani [93], reproposed the procedure previously described by Baek et al. in the year 1996 [110] (Scheme 13).

Briefly, CBG was prepared reacting olivetol dissolved in dry chloroform (CHCl_3_) with geraniol, in the presence of PTSA for 12 h at room temperature. The crude CBG was achieved as an oil, which was purified by silica gel chromatographic column. CBG was isolated with a low 15% yield.

#### 4.2.3. Synthesis of (−)-CBD

The first synthetic routes available to synthetize (−)-CBD [114,115,116,117] are of scarce practical value, as they lead to (−)-CBD in mediocre or even insignificant yields and the unnatural CBD isomer (Abn-CBD) (see Figure 11) was obtained in amounts considerably larger than those of (−)-CBD. Despite such poor results, the procedure proposed by Petrzilka et al. in the year 1969, but using *E*-(+)*-p*-mentha-2,8-dien-1-ol in place of *Z*-(+)*-p*-mentha-2,8-dien-1-ol and olivetol in benzene in the presence of catalytic amounts of PTSA was reproduced by Papahatjis et al. in the year 2002, achieving (−)-CBD in 31% yield [118]. The best route to (−)-CBD described is the condensation of (+)*-p*-mentha-diene-l-olo with olivetol in the presence of weak acids, reported by Razdan et al. in the year 1974 and Uliss et al. the next year [117,119]. In this case, the Abn-CBD obtained was converted to (−)-CBD with BF_3_-etherate by a retro-Friedel-Crafts reaction, followed by recombination. However, with this reagent the reaction proceeded further causing cyclisation of (−)-CBD.

**Scheme 14:**
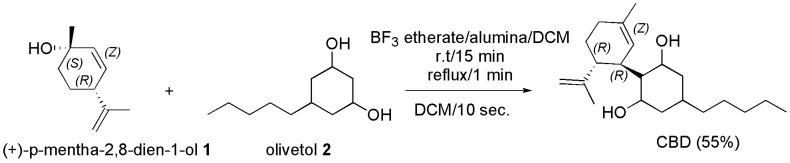
Synthesis of (−)-CBD [108]. Alumina = Al_2_O_3_.

In the year 1985, it was reported that when BF_3_-etherate on alumina is used as condensing agent in the reaction of *Z*-(+)*-p*-mentha-2, 8-dien-1-ol (**1**) with olivetol (**2**), (−)-CBD was obtained as the major product, in 55% yield as chromatographically pure oil, or in 41% yield as crystalline material (Scheme 14) [108].

No cyclization was observed, and side products were much more polar (14% yield) or much less polar (6% yield) than (−)-CBD.

Particularly, BF_3_-etherate was added under nitrogen to a stirred suspension of basic aluminum oxide (Al_2_O_3_) in dry DCM, and after 15 min at room temperature and 1 min at 40–41 °C, (+)*-p*-mentha-2,8-dien-l-ol and olivetol dissolved in DCM were added. The reaction was quenched within 10 s with 10% aqueous solution of sodium bicarbonate (10 mL), and after evaporation of the organic extracts, (−)-CBD was obtained.

**Scheme 15:**
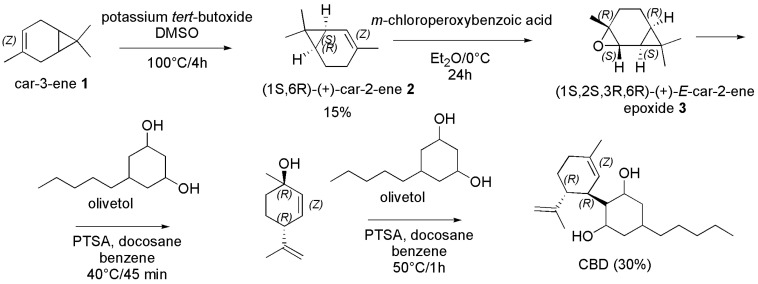
Synthesis of (−)-CBD [120].

In 1988, Crombie et al. [120] reported the reaction of (1S,2S,3R,6R)-(+)-*E*-car-2-ene epoxide and that of *p*-menthadienol with olivetol in the presence of PTSA. Particularly, the starting material (1S,2S,3R,6R)-(+)-*E*-car-2-ene epoxide (**3**) was prepared starting from car-3-ene (**1**) by its treatment with potassium *tert*-butoxide to achieve the derivative **2**, which after epoxidation with *m*-chloroperoxybenzoic acid provided the desired compound **3** (Scheme 15). Otherwise, *p*-menthadienol was prepared by citral with HCl in water or with PTSA in water/DCM (reaction not reported). The authors observed that in both cases, among other minor compounds, (−)-CBD, Abn-CBD, 1-THC and 6-THC were obtained. In particular, when (1S,2S,3R,6R)-(+)-*E*-car-2-ene epoxide was reacted with olivetol in benzene in the presence of docosane as catalyst for 45 min at 40 °C, the main product which formed was *p*-menthadienol with traces of (−)-CBD, Abn-CBD and THC. The further reaction of the obtained *p*-menthadienol with olivetol for 1h at 50 °C in benzene with docosane as well, afforded (−)-CBD in 30% yield, together with THC (18%), Δ^8^-THC (6%), and Abn-CBD (13%) (Scheme 15).

Rationally, the direct reaction of *p*-menthadienol (**1**) with olivetol in the conditions above reported afforded (−)-CBD in 30% yield (last part of Scheme 15).

**Scheme 16:**
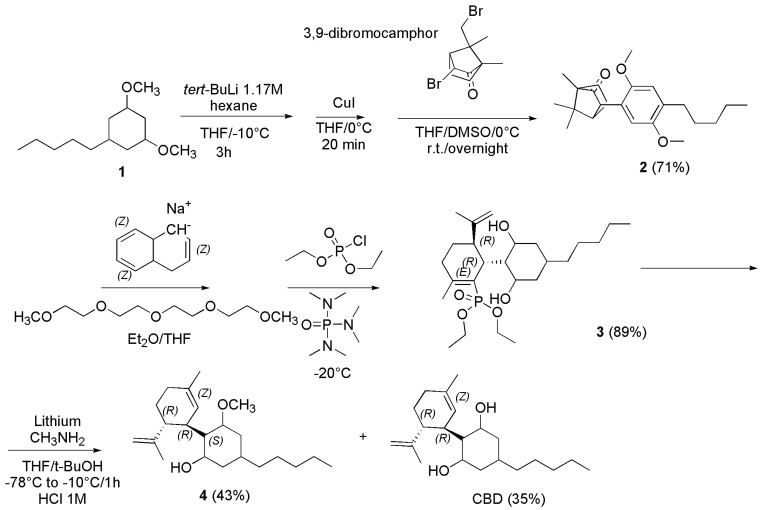
Synthesis of (−)-CBD [123].

Although in different conditions in terms of solvents, times, temperature, stereochemistry and catalysts, the reaction of *p*-menthadienol with olivetol was exploited by different research groups. Kinney et al. reacted *E*-(+)-*p*-mentha-2, 8-dien-1-ol with olivetol in toluene with PTSA for 1.5 h at 18–25 °C achieving (−)-CBD in 20% yield [121]. Also, Villano et al. in the year 2022 condensed olivetol with commercially available *Z*-(+)*-p*-mentha-2, 8-dien-1-ol in the presence of 33 mol% of wet PTSA in toluene at 0 °C for 3 h, affording a mixture of normal (−)-CBD and Abn-CBD, which were isolated in 26% and 38% yield respectively after a chromatographic column. Importantly, under these experimental conditions, no tricyclic structure was produced [122]. A different synthetic procedure was reported by Vaillancourt et al. in the year 1992 [123]. The authors described a new synthesis of (−)-CBD via the α-arylation of camphor, achieving both (−)-CBD and (−)-CBD mono methyl ether as shown in Scheme 16 [123].

Particularly, the authors prepared the endo-3-(2,5-dimethoxy-4-*n*-pentylphenyl)camphor (**2**), by reacting first olivetol dimethyl ether (**2**) dissolved in dry THF under nitrogen at −10 °C with *tert*-butyllithium (*tert*-BuLi) 1.7 M in hexanes for 3 h under stirring. Then the solution obtained was transferred to a solution of CuI in dry THF at 0 °C and the mixture was stirred for 20 min. Upon dilution with DMSO, the obtained solution was transferred dropwise to a solution of 3,9-dibromocamphor dissolved in dry THF/DMSO at 0 °C, and the reaction was then allowed to warm to room temperature and was stirred overnight. After the proper work-up and removal of the solvent in vacuo, the crude product was chromatographed and recrystallized from EtOH to achieve **2** in 71% yield. Compound **2** was transformed into the vinyl phosphate derivative **3**, by dissolving it in dry THF under nitrogen and titrating the obtained solution cooled to −78 °C with a freshly prepared 0.4 M Na-naphthalenide/0.4 M tetra-ethyleneglycol dimethyl ether solution (see later) in THF until a deep green color persisted. The green mixture was then added with diethyl chlorophosphate and hexamethylfosforamide (HMPA), was allowed to warm to −20 °C, and opportunely treated to provide the crude product which was subjected to a short silica column obtaining the desired enol phosphate **3** as a colorless oil (89%). (−)-CBD and (−)-CBD monomethyl ether (**4**), were finally obtained by adding the vinyl phosphate **3** dissolved in dry THF and *tert*-butanol (*t*-BuOH) to an excess of lithium foil in methylamine (MeNH_2_) at −78 °C. When addition was complete, the reaction was allowed to stir at −10 °C for 1 h and treated by acidification with HCl 1M. After extraction and removal of organic solvent the crude reaction mixture was chromatographed on neutral alumina, achieving (−)-CBD monomethyl ether in 43% yield. Further elution afforded (−)-CBD in 35% yield. The 0.4 M Na-naphthalenide/0.4 M tetra-ethylene glycol dimethyl ether solution in THF was prepared adding naphthalene in dry THF with sodium metal. The mixture was allowed to stir for 2 h, and then 2.41 mL of tetra-ethylene glycol dimethyl ether was added. The mixture was allowed to stir an additional hour at room temperature before use (Scheme not reported).

**Scheme 17:**
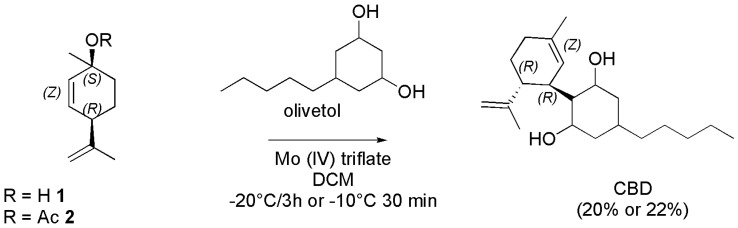
Synthesis of (−)-CBD [124].

Later in the year 2002, Malkov et al. described the synthesis of (−)-CBD in 14–22% yield using *Z*-(+)-*p*-mentha-2, 8-dien-1-ol (**1**) or its acetate derivative **2** and olivetol in dichloromethane (DCM) and molybdenum catalysts (Scheme 17) [124].

**Scheme 18:**
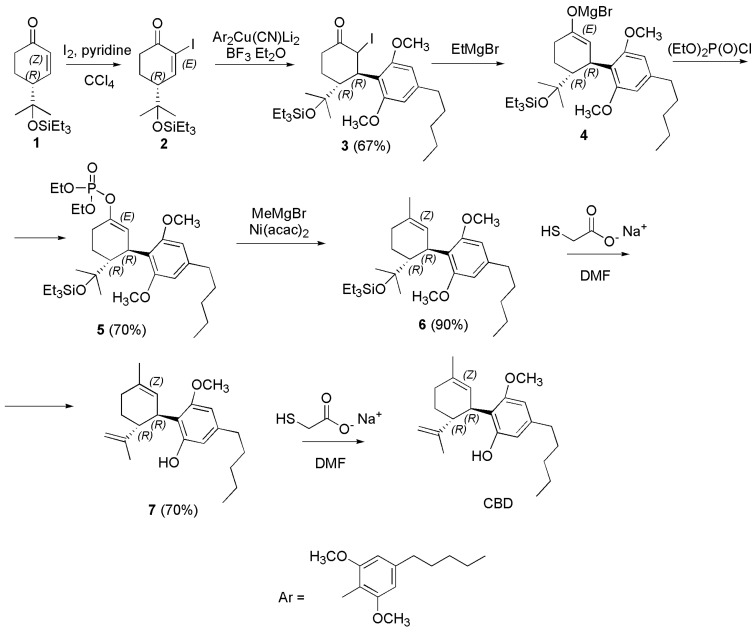
Synthesis of (−)-CBD [125]. Ni(acac)_2_ = nickel (II) acetylacetonate.

According to the reported results, starting from *Z*-(+)-*p*-mentha-2, 8-dien-1-ol **(1)** and olivetol dissolved in DCM and using the molybdenum Mo (IV) triflate complex as catalyst at −20 °C for 3 h (−)-CBD was obtained in 20% yield. Similar results (22% yield) were obtained starting from *Z*-(+)-*p*-mentha-2, 8-dien-1-ol acetate (**2**), olivetol and the same catalyst, and stirring the reaction mixture at −10 °C for 30 min. On the contrary, with the bimetallic Mo (II) catalyst V, and stirring **2** and olivetol at 20 °C for 4 h (−)-CBD was obtained in a lower yield (14%). Kobayashi et al. in the year 2001 reported the BF_3_-promoted 1,4-addition of bulky aryl groups, including dimethoxy olivetol, to an α-iodo enone (**2**), prepared from the parent enone (**1**), thus affording a β-aryl-α-iodo ketone derivative (**3**). Its subsequent reaction with EtMgBr furnished the magnesium enolate (**4**), which upon reactions with ClP(O)(OEt)_2_ gave an enol phosphate (**5**), which was applied successfully to the synthesis of (−)-CBD (Scheme 18) [125].

Particularly, the enone **1** was converted to the α-iodocyclohexenone (**2**) with I_2_ and pyridine in CCl_4_ with good yield. The 1,4-addition of Ar_2_Cu(CN)Li_2_ to **2** promoted by BF_3_·OEt_2_ furnished ketone **3** in 67% yield, after aqueous workup and purification by chromatography. Then, EtMgBr was successful used to generate the corresponding enolate **4**, which provided the enol phosphate **5** by reaction with (EtO)_2_P(O)Cl in 70% yield (Scheme 18). Methylation of **5** with MeMgBr in the presence of nickel (II) acetylacetonate (Ni(acac)_2_) afforded **6**, and its subsequent exposure to sodium ethyl thiolate (EtSNa) in DMF resulted in the deprotection of the triethylsilil (TES) group and of one of the MeO groups to furnish **7**. Attempted one-step deprotection of the two MeO groups under more vigorous conditions was unsuccessful, therefore **7** was converted in (−)-CBD by further exposure to EtSNa in DMF.

**Scheme 19:**
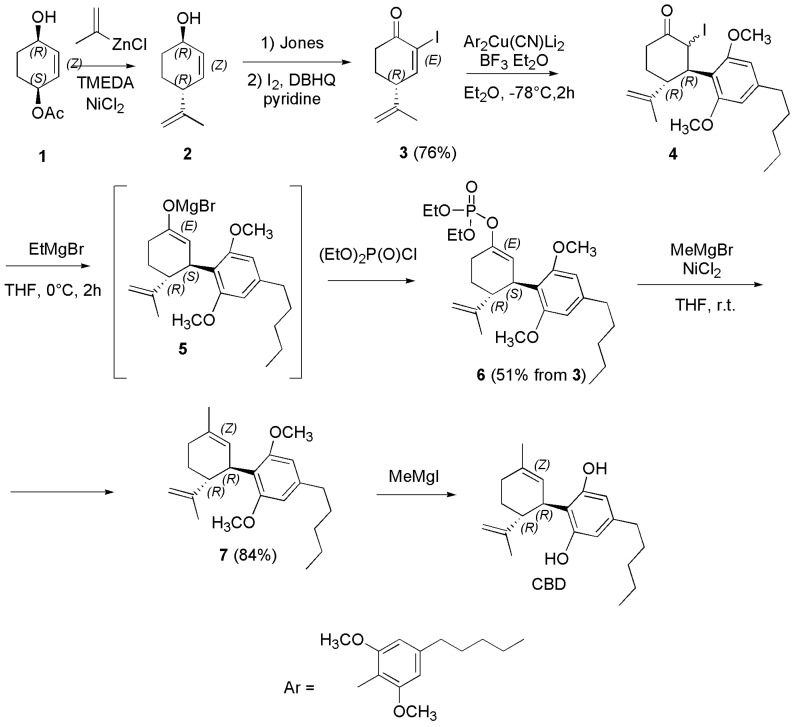
Synthesis of (−)-CBD [126].

Later in 2006, the same group reported a new reagent system for synthesizing (−)-CBD and its analogues via alkenylation of cyclohexenyl diol monoacetate according to Scheme 19 [126].

**Scheme 20:**
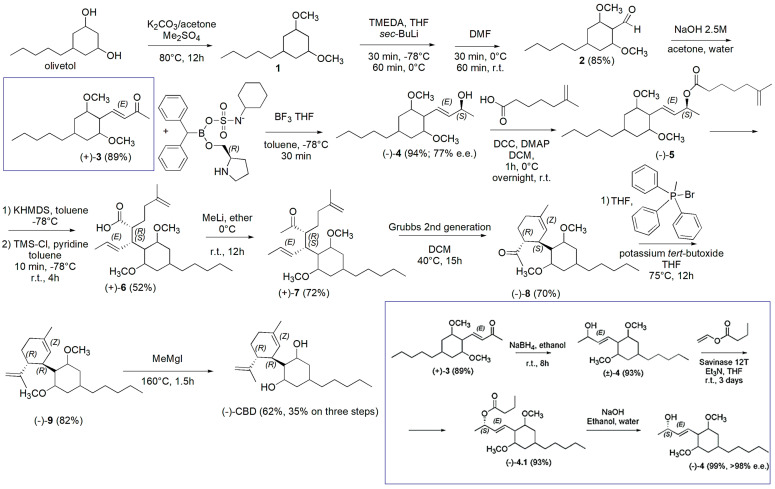
Synthesis of (−)-CBD [127]. In the blue square the alternative route described in the main text starting from **3** has been reported.

Briefly, by a nickel-catalyzed allylation of 2-cyclohexene-1,4-diol monoacetate (**1**) with a new reagent consisting of (alkenyl)ZnCl/TMEDA, the SN2-type product, namely *E*-(+)-*p*-mentha-2,8-dien-1-ol (**2**) was achieved with 94% regioselectivity in good yield. Oxidation of **2** afforded the intermediate enone, which underwent iodination at the α position by I_2_ in the presence of 2,5-di-*tert*-butylhydroquinone (DBHQ) as a radical scavenger to produce the α-iodo enone (**3**) in 63% yield (two steps). Addition of the 2,6-dimethoxy-4-pentylphenyl group of olivetol (abbreviated as Ar in the first part of the Scheme) to **3** was performed with the higher-order cyanocuprate derivative (Ar_2_Cu(CN)Li_2_) in turn synthesized from the Aryl (Ar) lithium anion and CuCN (not reported), obtaining compound **4**, as a 1:1 stereoisomeric mixture at the α position. Compound **4** underwent reaction with EtMgBr to produce the reactive magnesium enolate **5**, which was quenched with ClP(O)(OEt)_2_ to furnish enol phosphate **6** in 51% yield from **3**. Nickel-catalyzed coupling of **6** with MeMgCl afforded dimethyl ether **7** in good yield. Finally, (−)-CBD was obtained upon demethylation of **7** using MeMgI. Zachary et al. in the year 2018 reported a practical synthetic approach to synthetize Δ^9^-THC, and (−)-CBD. Particularly, (−)-CBD was synthesized according to Scheme 20 [127].

Briefly, olivetol and K_2_CO_3_ in acetone were added with dimethyl sulphate (Me_2_SO_4_) in 5 min at room temperature and then the mixture was heated to 80 °C for 12 h under argon, achieving the crude olivetol dimethyl ether (**1**) as an oil which was purified by column chromatography (98% yield). A yellow solution of **1** and TMEDA in anhydrous THF at −78 °C under argon was added with *sec*-butyllithium (*sec*-BuLi) and was stirred for 30 min at −78 °C and for 60 min at 0 °C, before being added with anhydrous DMF. The mixture was stirred at 0 °C for 30 min and for additional 60 min at room temperature. Upon proper work up and silica gel column chromatography the pure aldehyde derivative **2** was obtained as a yellow oil in 85% yield. Aldehyde **2** was converted in the enone **3** via an aldolic condensation with acetone in water using a 2.5M NaOH solution and heating the reaction mixture to 60 °C for 12 h (89% yield). The carbonylic group of **3** was reduced in toluene at −78 °C under argon, using a solution of (R)-CBS oxazaborolidine ligand (see Scheme 20) and BH_3_•THF complex. The reaction mixture continued to stir for 30 min at −78 °C obtaining the crude product (−)-**4** which was further purified by silica gel column chromatography achieving the pure compound (−)-**4** in 94% yield (77% enantiomeric excess (e.e.)) as a clear colorless oil that solidified upon standing. An alternative to generate a product with high enantiopurity consisted of an enzymatic approach using an inexpensive and readily available enzyme. In this regard, compound **3** in the blue square was reduced with sodium borohydride (NaBH_4_) affording the racemic alcohol (±)-**4**, which was acylated with vinyl butyrate in the presence of Savinase 12T thus providing the ester (−)-**4.1** which was hydrolyzed with NaOH affording (−)-**4** with >98% e.e. in 38% overall yield for the three steps (blue square in Scheme 20).

Compound (−)-**4** was converted in the carboxylate (−)-**5** by its acylation with 5-methyl-5-hexencarboxylic acid in DCM in the presence of DCC and DMAP. After 1 h stirring at 0 °C and then overnight at room temperature, (−)-**5** was achieved as crude material which was purified by column chromatography. Compound (−)-**5** was treated with KHMDS in anhydrous toluene at −78 °C for 1 h, then a solution of anhydrous pyridine and tetramethylsilyl chloride (TMS-Cl) in anhydrous toluene was added and the mixture was stirred at −78 °C for 10 min and at room temperature for an additional 4 h. Upon the Ireland−Claisen rearrangement, compound (+)-**6** was achieved as a white crystalline solid that could be recrystallized using hexanes in a 52% overall yield. Treatment of (+)-**6** in ether at 0 °C with methyllithium (MeLi) and stirring overnight at room temperature led to the formation of ketone (+)-**7** as colorless oil in 71.8% yield, after chromatography column. Compound (+)-**7** could be cyclized and then converted into (−)-CBD, via Wittig methylenation and deprotection. Particularly, compound (+)-**7** and Grubbs 2nd generation catalyst in DCM were first stirred for a total of 15 h at 40 °C, thus achieving compound (−)-**8** in 69.6% yield. Then, by reaction at room temperature of (−)-**8** with bromo(methyl)triphenylphosphorane in THF, followed by the addition of potassium *tert*-butoxide and stirring at 75 °C for 12 h, compound (−)-**9** was isolated in 82% yield. After its demethylated in anhydrous ether under argon with MeMgI and heating to 160 °C for 1.5 h, (−)-CBD was obtained in 62% (35% on three steps) yield as a light-yellow oil.

**Scheme 21:**
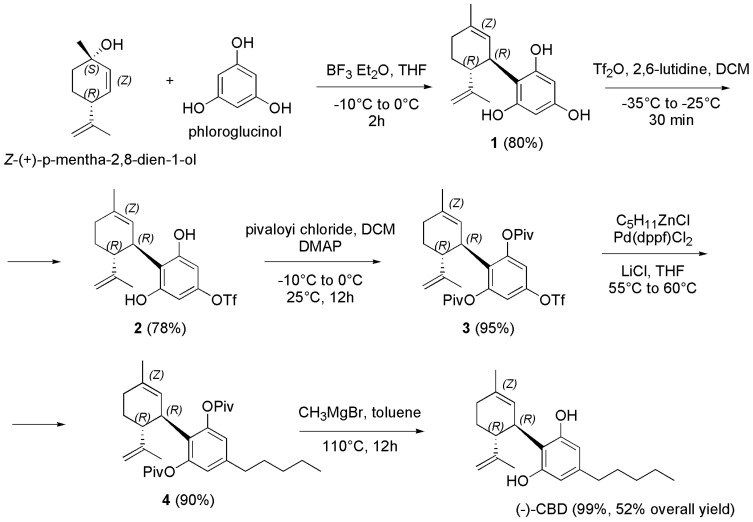
Synthesis of (−)-CBD [128].

In the year 2020, Gong et al. reported a novel synthetic procedure for making (−)-CBD on a 10 g scale, by a late-stage diversification method, starting from commercially available phloroglucinol. First, the key intermediate (−)-CBD-2OPiv-OTf was achieved which underwent Negishi cross-coupling with the pentyl chain to give (−)-CBD in 52% overall yield. By this approach using the symmetric phloroglucinol the generation of positional isomers (Abn-CBDs) was avoided (Scheme 21) [128].

Briefly, by a Friedel–Crafts alkylation of phloroglucinol with *Z*-(+)-*p*-mentha-2,8-dien-1-ol in a ratio of 1:10 in presence of BF_3_ etherate gave the desired product **1** in an excellent 80% yield. By treatment of **1** with trifluoromethanesulfonic anhydride (Tf_2_O) in the presence of 2,6-lutidine at −30 to −20 °C in DCM using 1.5 equivalent of **1**, to prevent double triflation, afforded the triflate derivative **2**, which was isolated by silica gel column chromatography in a 78.1% yield. Compound **2** was treated with a solution of pivaloyl chloride (Piv-Cl) in DCM and a solution of DMAP in pyridine at −10 to 0 °C. Subsequently, the mixture was stirred at 25 °C for 12 h, to obtain the O-protected derivative **3** in 95% yield after column chromatography as a yellow oil. Pentyl zinc chloride (C_5_H_11_ZnCl), was prepared in one step by the transmetalation of the correspondent Grignard C_5_H_11_MgBr. Particularly, anhydrous zinc chloride and anhydrous lithium chloride were dissolved in anhydrous THF and cooled to −10 °C, added with C_5_H_11_MgBr and stirred first at −10 °C for 15 min and then at room temperature for 1.5 h. The obtained mixture was added with a solution of **3** in THF and then with [1,1′-Bis(diphenylphosphino)ferrocene]dichloropalladium(II) (Pd(dppf)Cl_2_), as cross-coupling agent, to provide **4** in 90% yield after stirring at 55–60 °C for proper time and after column chromatography. (−)-CBD was finally obtained upon deprotection with MeMgBr (3 M in Et_2_O) in toluene at 110 °C for 12 h in 99% yield after silica gel column chromatography.

**Scheme 22:**
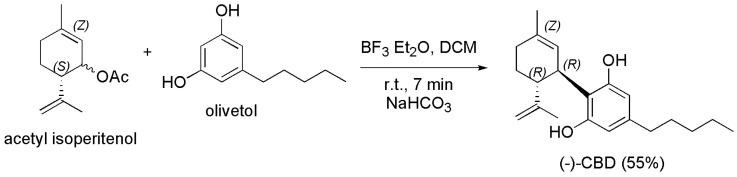
Synthesis of (−)-CBD [129].

Chiurchiu et al. in the year 2021 reported an innovative and high yielding continuous approach for producing (−)-CBD, strongly reducing its cyclization into THC (traces), thus achieving (−)-CBD in 55% yield. Particularly, by means of flow chemistry, and following their studies concerning the use of this technology for synthesizing highly functionalized materials, the authors inserted acetyl isoperitenol and olivetol dissolved in DCM into a reservoir A, while BF_3_·Et_2_O dissolved in DCM into a reservoir B. Subsequently, they pumped the two solutions simultaneously into a T-connector before passing through a 3 mL PTFE coil reactor (7 min residence time). The outgoing solution was dropped into a flask containing a stirring saturated solution of NaHCO_3_ from which (−)-CBD was extracted and purified by silica gel chromatography to provide pure (−)-CBD in 55% yield (Scheme 22) [129].

**Scheme 23:**
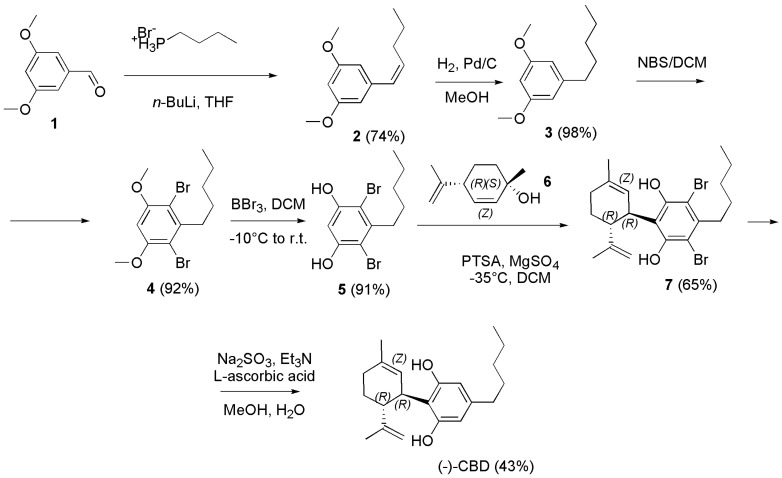
Synthesis of (−)-CBD [76].

The main by-products were Abn-CBD and the dialkylated cannabidiol recovered in 19 % and 4 % of yield respectively, THC was observed in traces (GC < 0.4 %) and its isolation was unfeasible. In the same year, Navarro et al. followed a practical approach to prepare (−)-CBD that avoided the formation of the side abnormal regio-isomers by using protected 4,6-dihalo-olivetol in coupling reaction as shown in Scheme 23 [76].

**Scheme 24:**
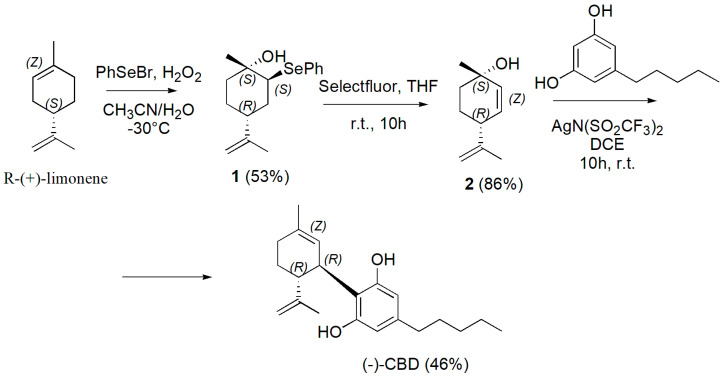
Synthesis of (−)-CBD [130].

Briefly, the synthesis began by the Wittig reaction of butyl phosphonium bromide with commercially available 3,5-dimethoxybenzaldehyde (**1**), to deliver the olefin derivative **2** as mixture of Z and *E* isomers, which was conveniently reduced with hydrogen under pressure in presence of Pd/C as catalyst to the olivetol dimethyl ether (**3**). Regioselective electrophilic aromatic bromination of **3** using 2.3 equivalents of N-bromosuccinimide (NBS) in DCM at room temperature produced exclusively the 4,6-dibrominated product **4** in good yield. Then, the methyl ether-protecting groups were removed with boron tribromide to generate the key resorcinol intermediate **5** which was submitted to the Friedel–Craft alkylation with (1S,4R)-4-isopropenyl-1-methyl-2-cyclohexen-1-ol (**6**) in DCM, in the presence of PTSA as catalyst, thus affording the adduct **7** as single diastereomer. Finally, reductive dehalogenation using sodium sulfite in the presence of triethylamine (Et_3_N) in a mixture of MeOH and H_2_O at 75 °C delivered the targeted cannabinoid (−)-CBD in 43% yield. Anand et al. in the year 2022, developed a three-step concise and stereoselective synthesis route to (−)-CBD and (+)-CBD, using inexpensive and readily available starting material, such as R-(+)-limonene and S-(−)-limonene respectively. The synthesis involved the diastereoselective bi-functionalization of limonene, followed by effective elimination leading to the generation of the key chiral (+)- or (−)-*p*-mentha-2,8-dien-1-ols. Such dienols on coupling with olivetol under silver bis (trifluoromethanesulfonyl) imide (AgN(SO_2_CF_3_)_2_) as catalysis provided regiospecific (−)-CBD or (+)-CBD in good yield (Scheme 24) [130].

**Scheme 25:**
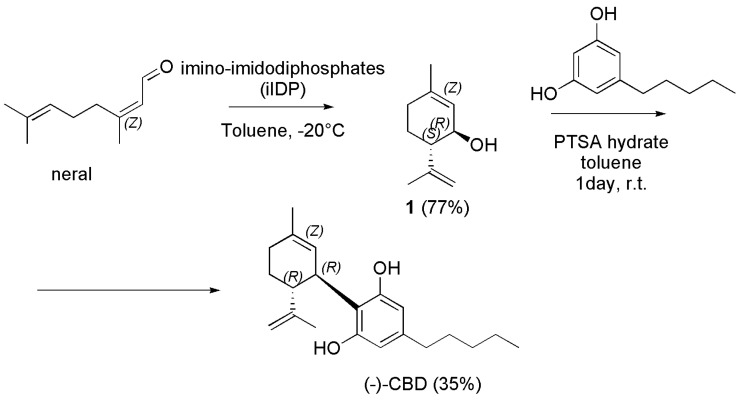
Synthesis of (−)-CBD [131].

Briefly, the present approach started with the direct generation of diastereoselective bi-functionalized 2-phenylseleninyl-*p*-menth-8-en-1-ol (**1**) from readily available and inexpensive starting material R-(+)-limonene. Particularly, electrophilic phenyl selenium bromide and H_2_O_2_ in a mixture acetonitrile/water at −30 °C were used, thus achieving compound **1** in 53% yield. The removal of SePh to synthesize (+)-menthadienol **2** was carried out using Selectfluor in THF at room temperature for 10 h achieving optically pure (+)-*p*-mentha-2,8-dien-1-ol **2** in 86% yield. Compound **2** was then coupled with olivetol in DCM using AgN(SO_2_CF_3_)_2_ at room temperature for 10 h achieving (−)-CBD in 46% yield. Similarly, starting from S-(−)-limonene, (+)-CBD was synthesized. Briefly, the synthesis began from commercially available S-(−)-limonene, which was subjected to stereoselective bifunctionalization and elimination cascade to afford (−)-*p*-mentha-2,8-dien-1-ol. Pleasingly, (−)-*p*-mentha-2,8-dien-1-ol on reaction with olivetol in the presence of AgN(SO_2_CF_3_)_2_ afforded the single isomer of (+)-CBD. The last synthetic procedure we report here to obtain (−)-CBD was very recently described by Grimm et al. (Scheme 25) [131].

Briefly, neral (*Z*-citral) was cyclized to isopiperitrol using imino-imidodiphosphates (iIDP), featuring a bifunctional inner-core system with an acidic P=NHTf moiety and a basic P=O moiety, thus combining excellent reactivity and selectivity, and furnishing (1R,6S)- *E*-isopiperitenol (**1**) in good yield (77%) and excellent diastereo- and enantioselectivity. Interestingly, such cyclization can be performed easily on a multigram scale (>4 g) without any loss of selectivity or yield, and catalyst can be recovered in excellent yield (95%) and re-used in further cyclization reactions. Then, direct access to (−)-CBD from isopiperitenol (**1**) and olivetol was provided in 35% yield under mild conditions using PTSA as catalyst in DCM at room temperature for 5 days. It is noteworthy that no further reaction of (−)-CBD to the corresponding THC was observed.

**Scheme 26:**
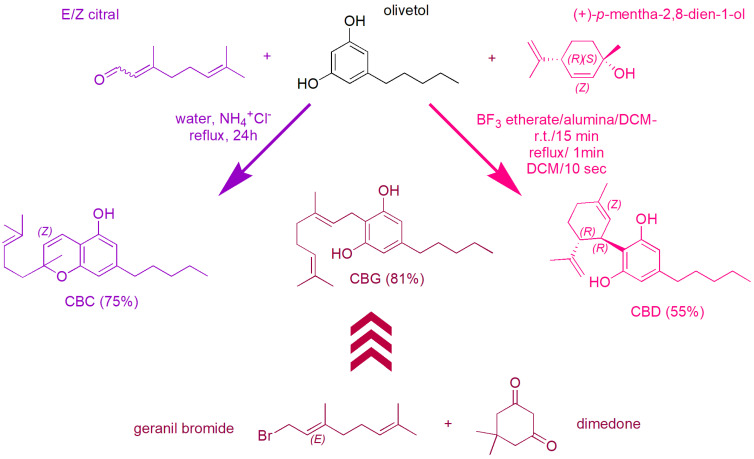
Overview scheme showing the synthetic procedures selected by us as the most convenient in terms of yields to obtain CBC (purple route), CBD (pink route) and CBG (amaranth route). In the route to CBG, each arrow represents a reaction step.

Following an overview scheme (Scheme 26) showing the synthetic procedures selected by us as the most convenient in terms of yields to obtain CBC (purple route) [103], CBD (pink route) [108] and CBG (amaranth route) [113]. Note that, while the synthetic paths leading to CBC and CBD are both one step processes shearing olivetol as reagent, that leading to CBG is a complicate multi-step process, using reagents completely different from those leading to CBC and CBD.

## 5. Conclusions and Future Perspectives

Here, cannabinoids have been reviewed in terms of classification, chemical structures, mode of action, SARs, main pharmacological properties, current applications, clinical applicability, and antimicrobial properties, according to what has been reported so far. In doing so, we have detected the most promising compounds for the development of new efficient antibacterial agents to counteract MDR pathogens, promising as therapeutic agents because deprived of the psychotropic side effects, typical of the well-known THC. From the scenario that arose from this overture, it emerged that CBC, CBG and CBD, known to exclusively possess beneficial non-mind-altering pharmacological effects and reported to have potent antibacterial effects, may be the best candidates for the development of new antibiotics. So, we have systematically reviewed the synthetic pathways utilized for their preparation, thus providing a rich pool of different synthetic procedures, which can enable chemists to produce consistent amounts of these therapeutically promising PCs. Gathering in a single work the experience gained over the years by several scientists in the CBC, CBG and CBD synthesis, this review can represent a sort of manual where synthetic chemists can choose the most suitable procedure to prepare them, also according to their resources. An extensive synthetic work supported by this review will afford the material necessary for further studies and will encourage the development of new PCs-based antibiotics. Additionally, although already very potent as such, we think that additional SAR studies, specifically on CBC, CBG and CBD, are needed for detecting how these natural compounds could be modified, in order to focalize the plethora of their pharmacological properties only towards the antibacterial one, thus increasing their potency. Moreover, we think that, since these molecules have demonstrated only poor activity on bacteria of Gram-negative species, which are the ones that most endanger public health, proper structural modification could help to enhance their activity on frightening species such as resistant *E. coli*, *P. aeruginosa*, *Klebsiella* supp., *Salmonella* supp. Etc. Surely, starting from synthetic compounds obtainable in good amounts following the procedures reported here, the preparation of new and specialized cannabinoids might help to further elucidate the biological mode of action of cannabinoids on bacteria, which has not been clarified so far. In fact, although it is recognized that cannabinoids act by impairing the cytoplasmic membrane of bacteria, the mode exploited to achieve this damage remains unveiled. Note that, although a large collection of synthetically prepared cannabinoids has been reported, and the synthetic approaches employed for preparing THC have been analytically reviewed by Bloemendal and colleagues recently, those employed to prepare the non-psychotropic CBC, CBG and CBD have not yet been systematically reviewed.

## Figures and Tables

**Table 1:** Strategies for combating antibiotic resistance.

Strategies for Combating Antibiotic Resistance		Ref.
Nanotechnology	Quality by design (QbD) approach	[13]
Computational methods	In silico modelling	[14]
	Fragment-based drug design (FBDD)	[15]
Antibiotic alternatives	Antimicrobial peptides (AMPs)	[12]
	Essential oils	
	Anti-Quorum Sensing (QS)	
	Darobactins	
	Vitamin B6	
	Bacteriophages	
	Odilorhabdins	
	18-β-glycyrrhetinic acid	
	Cannabinoids	
Drug reproposing	Ticagrelor	[16]
	Mitomycin C (MMC)	
	Auranofin	
	Pentamidine	
	Zidovudine (AZT)	
Synthesis of novel antibacterial agents	Lactones	[17]
	Piperidinol	[18]
	Sugar-based bactericides	[19]
	Isoxazole derivatives	[20]
	Carbazole	[21]
Prodrugs	Siderophores	[22]
	Carbapenem-oxazolidinones	
	Oral Gyrb/ParE dual binding inhibitor	
	AMPs prodrugs	
Development of efficient diagnostic agents (RDTs)	Point-of-care tests (POCTs) Molecular (genotyping) assays	[23]
Combination therapy	Penicillin with streptomycin * Rifampin–isoniazid–pyrazinamide ** Trimethoprim-sulfamethoxazole Quinupristin-dalfopristin Bacitracin-polymyxin B Bacitracin-polymyxin B-gramicidin Neomycin,-bacitracin-gramicidin	[24]
	β-Lactams antibiotics-β-Lactamase inhibitors ***	[25,26]
Awareness and knowledge of antibiotic prescribing		[27]

RDTs = rapid diagnostic tests; * For enterococcal infections; ** in the treatment of tuberculosis; *** ceftazidime/avibactam, meropenem/vaborbactam and imipenem/relebactam.

**Table 2:** Locations of CB1 and CB2 receptors in the human body.

Receptor Type	Location	Sublocation	Ref.
CB1	Central nervous system (CNS)	Hippocampus, cerebellum, basal ganglia, cortical regions Olfactory areas	[46]
	Peripheral nerve terminals Extra-neuronal sites	Eye, vascular endothelium, adipose tissue, lungs, liver Spleen, kidneys, uterus, prostate, testis, stomach, placenta Skeletal, muscles	
CB2	Peripheral immune system tissues	Spleen, tonsils, thymus, lymph nodes	[47]
	Peripheral immune system cells	B cells, natural killer cells, monocytes, macrophages Neutrophils, CD8+ T cells, CD4+ T cells	
	CNS *	Cerebellum, olfactory tubercle, striatum Thalamic nuclei (hippocampus and amygdala)	

* Under certain circumstances, most notably during inflammation.

**Table 3:** Selectivity of some SCs for CB1 and CB2 receptors, and their effects.

SCs	Binding Affinity		Effects	Refs.
	CB1 (Ki, nM)	CB2 (Ki, nM)		
Dronabinol *	15	51	Appetite stimulant Psychotropic effects Analgesic ↓ Nausea Antiemetic	[60]
Methanandamide (AM-356)	20	815		[61]
SR144528	280	0.1	Anti-inflammatory Analgesic ↓ Neuropathic pain	[48]
Cannabinor ^#^ (PRS-211,375)	5585	17.4		[62]
CP47,597	2.1 (Kd)	56	Analgesic	[48]
JTE907	490	2.2	Anti-inflammatory	[48]
JWH133	680	3.4	↓↓ Neurotoxicity Anti-inflammatory ↓ Alzheimer symptoms	[48]
AM1241	272	3.4	Analgesic effects ↓ Hyperalgesia ↓ Allodynia Amyotrophic lateral sclerosis	[48]
GW405,833	8640	7.2	↓ Hyperalgesia ↓ Allodynia	[48]
L-759,633	1604	9.8	Analgesic Antianxiety Antidepressant Anti-inflammatory ↓ Alzheimer symptoms	[48]
JWH139	2290	14		
HU308	115,000	23		
AM630	3795	32		
HU-210	0.061	0.52		[63]
L-759,656	4888	↑11.8		[64]
WIN 55,212-2	1.9		Analgesic Anti-inflammatory ↓ Alzheimer symptoms	[65]
JWH015	383	13.8	Analgesic Anti-inflammatory	[66]
WIN 48,098 (Pravadoline)	4.9 (IC_50_)		Analgesic Anti-inflammatory	[67]

Ki = Defined kinetically as the ratio of rate constants koff/kon for the binding of a ligand to the receptor. This is the same as Kd; IC_50_ = the concentration of ligand required to saturate half of the receptor; * Approved by the FDA as safe and effective for HIV/AIDS-induced anorexia and chemotherapy-induced nausea and vomiting only; ^#^ failed in Phase IIb human clinical trials due to lack of efficacy; ↓ = reduction of; ↓↓ = strong reduction of.

**Table 4:** The most relevant cannabinoids which were in the past or recently assayed to assess their antimicrobial properties.

Precannabinoids	Cannabinoids	Synthetic Compounds
Cannabichromenic acid (CBCA)	Cannabichromene (CBC) *	Δ^11^-THC **^S^ Me-CBD Me-CBG Ac-CBD Ac-CBG PhEO-CBD PhEO-CBG MeO-CBD MeO-CBG Abn-CBD Abn-CBG Carmagerol °° BP-CBD CBC isomer (ICBC) CBC-C_o_ CBC-C_1_ ICBC-C_o_
Cannabidiolic acid (CBDA)	Cannabidiol (CBD) *	
Cannabigerolic acid (CBGA)	Cannabigerol (CBG) *	
Δ^9^-Tetrahydrocannabinolic acid A (THCAA)	Δ^9^-Tetrahydrocannabinol (Δ^9^-THC) *	
Δ^9^-Tetrahydrocannabinolic acid B (THCAB)		
Δ^9^-tetrahydrocannabivarin acid (THCVA)	Δ^9^-tetrahydrocannabivarin (THCV) *	
Cannabidivarinic acid (CBDVA)	Cannabidivarin (CBDV) *	
N.T.	Δ^8^-THC **^N^	
	Cannabicyclol (CBL) ^#^	
	(±)11-NCTHC °	
	(±)11-HTHC ^§^	
	Anantamide (ANA) ***^,$^	
	Arachidonyl serine (AraS) ^$^	

* From decarboxylation of acidic pre-cannabinoids; N.T. = not tested; **^N^ natural isomer of Δ^9^-THC; **^S^ synthetic isomer of Δ^9^-THC; Me-CBD = mono- di-methylated derivatives of CBD (Figure 11); Me-CBG = mono- di-methylated derivatives of CBG (Figure 11); Ac-CBD = acetylated derivative of CBD (Figure 11); Ac-CBG = acetylated derivative of CBG (Figure 11); PhEO-CBD = phenyl-ethyl ester of CBD (Figure 11); PhEO-CBG = phenyl-ethyl ester of CBG (Figure 11); MeO-CBD = methyl ester of CBD (Figure 11); MeO-CBG = methyl ester of CBG (Figure 11); Abn-CBD = abnormal (*orto*-isomer) CBD (Figure 11); Abn-CBG = abnormal (*orto*-isomer) CBG (Figure 11); °° polar analogue of CBG (Figure 11); BP-CBD = bis-prenylated analogue of CBD (Figure 11); ICBC, CBC-C_o_, CBC-C_1_, ICBC-C_o_ = isomers and analogous of CBC whose structures are available in Figure 11; ^#^ degradative product of CBC; ° not active secondary metabolite of THC which is formed in the body after cannabis is consumed; ^§^ main active metabolite of THC, which is formed in the body after Δ^9^-THC is consumed; *** also known as N-arachidonoyl-ethanolamine (AEA); ^$^ ECs.

**Table 5:** Antimicrobial effects against bacteria and fungi reported in the past and recently for several cannabinoids expressed as MICs (µg/mL) if not differently specified.

Compounds	Pathogens	MIC * (µg/mL)	Ref.	Comments
Δ^9^-THC	*S. aureus*	1–5	[82]	Binding to plasma proteins ↓ Activity on Gram-negative Psychotropic
	*Streptococcus pyogenes*	5		
	*S. milleri*	2		
	*Enterococcus faecalis*	5		
	*S. aureus*	1	[83]	
	EMRSA	0.5–2		
	*S. aureus* SA-1199B	2		
	*S. aureus* RN-4220	1		
	*S. aureus* XU212	1		
	MRSA USA300	2	[33]	
Δ^9^-THC acid A (THCAA)	MRSA USA300	4	[33]	Binding to plasma proteins ↓ Activity on Gram-negative Non-psychotropic ↑ Therapeutic potential ↑ Effects without the carboxylate moiety
	*S. aureus*	4	[83]	
	EMRSA	4–8		
	*S. aureus* SA-1199B	8		
	*S. aureus* RN-4220	4		
	*S. aureus* XU212	8		
Δ^8^-THC	MRSA USA300	2	[33]	Binding to plasma proteins ↓ Activity on Gram-negative Psychotropic
Δ^11^-THC	MRSA USA300	2	[33]	
Δ^9^-THCV	MRSA USA300	4	[33]	Lack psychotropic effects ↑ Therapeutic potential ↓ Activity on Gram-negative ↑ Effects without the carboxylate moiety
THCV acid (THCVA)	MRSA USA300	16	[33]	
CBD	*S. aureus*	1	[84]	Binding to plasma proteins ↓ Activity on Gram-negative Antiepileptic Anti-inflammatory Non-psychotropic ↑ Therapeutic potential
		1–5	[82]	
		0.5	[83]	
		1.25	[85]	
	EMRSA	1	[83]	
	*S. aureus* SA-1199B			
	*S. aureus* RN-4220			
	*S. aureus* XU212			
	MRSA USA300	2	[33]	
		4	[86]	
	*S. epidermidis*	2	[84]	
	*S. pyogenes*	2	[82]	
	*S. milleri*	1		
	*E. faecalis*	5		
	MRSE	4	[86]	
	*Listeria monocytogenes*	4		
	*E. faecalis*	8		
	*E. coli*	1.25	[85]	
CBN	MRSA USA300	2	[33]	Binding to plasma proteins ↓ Activity on Gram-negative Weakly psychotropic
	*S. aureus*	1	[83]	
	EMRSA			
	*S. aureus* SA-1199B			
	*S. aureus* RN-4220			
	*S. aureus* XU212			
Abn-CBD	*S. aureus*	1	[83]	Binding to plasma proteins ↓ Activity on Gram-negative Non-psychotropic ↑ Therapeutic potential
	EMRSA			
	*S. aureus* SA-1199B			
	*S. aureus* RN-4220			
	*S. aureus* XU212			
CBC	*B. subtilis*	0.39	[87]	↓ Activity on Gram-negative Non-psychotropic ↑ Therapeutic potential In case of acid compounds: ↑ Effects without the carboxylate moiety
	*S. aureus*	1.56		
	*Mycobacterium smegmatis*	12.5		
	*Candida albicans*	N.T.		
	*Saccharomyces cerevisiae*	25		
	*Trichophyton mentagrophytes*	25		
	MRSA USA300	8	[33]	
	EMRSA	2	[83]	
	*S. aureus*	2		
	*S. aureus* SA-1199B	2		
	*S. aureus* RN-4220	2		
	*S. aureus* XU212	1		
CBCA	MRSA USA300	2	[33]	
CBC isomer (ICBC)	*B. subtilis*	0.78	[87]	
	*S. aureus*	N.T.		
	*M. smegmatis*	25		
	*C. albicans*	50		
	*S. cerevisiae*	N.T.		
	*T. mentagrophytes*	N.T.		
CBC-C_o_	*B. subtilis*	6.25		
	*S. aureus*	12.5		
	*M. smegmatis*	12.5		
	*C. albicans*	50		
	*S. cerevisiae*	25		
	*T. mentagrophytes*	25		
CBC-C_1_	*B. subtilis*	3.12		
	*S. aureus*	3.12		
	*M. smegmatis*	3.12		
	*C. albicans*	N.T.		
	*S. cerevisiae*	6.25		
	*T. mentagrophytes*	6.25		
ICBC-C_o_	*B. subtilis*	6.25		
	*S. aureus*	12.5		
	*M. smegmatis*	12.5		
	*C. albicans*	12.5		
	*S. cerevisiae*	N.T.		
	*T. mentagrophytes*	6.25		
CBDA	*S. aureus*	2	[83]	↑ Effects without the carboxylate moiety ↓ Activity on Gram-negative Non-psychotropic ↑ Therapeutic potential ↑ Effects without the carboxylate moiety
	EMRSA			
	*S. aureus* SA-1199B			
	*S. aureus* RN-4220			
	*S. aureus* XU212			
	*S. epidermidis*	4	[84]	
	*S. aureus*	2		
	MRSA USA300	16	[33]	
		4	[84]	
CBGA	MRSA USA300	4	[33]	Non-psychotropic ↑ Therapeutic potential ↑ Effects without the carboxylate moiety ↓ Activity on Gram-negative
	EMRSA	2–4	[83]	
	*S. aureus*	4		
	*S. aureus* SA-1199B	4		
	*S. aureus* RN-4220	2		
	*S. aureus* XU212	4		
CBG	*Streptococcus mutans*	2.5	[88]	Non-psychotropic ↑ Membrane permeability Cause membrane hyperpolarization ↓ Membrane fluidity Non-psychotropic ↑ Therapeutic potential ↓ Activity on Gram-negative
	*S. sanguis*	1		
	*S. sobrinos*	5		
	*S. salivarius*	5		
	MRSA USA300	2	[33]	
	*S. aureus*	1	[83]	
	EMRSA	1–2		
	*S. aureus* SA-1199B	1		
	*S. aureus* RN-4220	1		
	*S. aureus* XU212	1		
	*C. albicans*	3 ^a^; (4) ^b^	[89]	
	*S. cerevisiae*	6 ^a^; (2) ^b^		
	*T. mentagrophytes*	5 ^a^; (4) ^b^		
Abn-CBG	*S. aureus*	1	[83]	
	EMRSA	2		
	*S. aureus* SA-1199B	2		
	*S. aureus* RN-4220	1		
	*S. aureus* XU212	0.5		
CBDV	MRSA USA300	8	[33]	Non-psychotropic ↑ Therapeutic potential ↓ Activity on Gram-negative
CBDVA	MRSA USA300	32	[33]	Non-psychotropic ↑ Therapeutic potential ↑ Effects without the carboxylate moiety ↓ Activity on Gram-negative
CBL	MRSA USA300	>32	[33]	Non-psychotropic ↑ Therapeutic potential
(±) 11-NCTHC				
(±) 11-HTHC	MRSA USA300	>32	[33]	Psychotropic
ANA	MRSA	>256 **	[90]	Psychotropic ↓ Membrane potential in bacteria ↓ Bacteria adhesion capacity ↓ Cells aggregation capacity Not bactericidal ↓ Activity on Gram-negative
		64 *** (51–54) ^#^		
AraS	MRSA	32->256 **	[90]	Psychotropic Neuroprotective ↓ Activity on Gram-negative Affect membrane potential in bacteria ↓ Bacteria adhesion capacity ↓ Cells aggregation capacity Not bactericidal
		64 *** (33–61) ^#^		

* After 24 h exposure on bacteria, after 48 h exposure on fungi; ^a^ activity was recorded as the width (in millimeters) of the inhibition zone measured from the edge of the agar well to the edge of the inhibition zone; ^b^ inhibition zone of Amphotericin B; ** on planktonic cells; *** on biofilm; # percentage of biofilm eradication; SA-1199B = fluoroquinolones-resistant; RN-4220 = macrolides-resistant; XU212 = tetracycline-resistant; MRSE = methicillin-resistant *S. epidermidis; S. aureus* = ATCC25923 strains; EMRSA = the major epidemic methicillin-resistant *S. aureus* occurring in U.K. hospitals; ↓ = low, lower, minor; ↑ = high, higher, strong.

## Data Availability

All material and data related to this study is already available in the main text.
